# Research on multi-person collaborative design of BIM drawing based on blockchain

**DOI:** 10.1038/s41598-022-20321-5

**Published:** 2022-09-29

**Authors:** Jinlong Wang, Yumin Shen, Xiaoyun Xiong, Xu Wang, Xiaoxue Fang

**Affiliations:** School of Information and Control Engineering, Qingdao University of Technology, Qingdao, Shandong China

**Keywords:** Electrical and electronic engineering, Computer science

## Abstract

The existing multi-person collaborative design scheme of Building Information Modeling (BIM) integrated with blockchain faces problems such as poor reliability of BIM drawing, inconsistent drawing information, redundant information, and inaccurate protection of copyright interests. This paper proposes a multi-person collaborative design model for BIM drawing that combines blockchain and InterPlanetary File System (IPFS). This model uses blockchain to store drawing design information to protect the copyright interests of designers and combines IPFS to ensure the reliability of drawing. A cycle division mechanism is designed to solve the problem of drawing information synchronization when multiple people collaborate in design. The Semantic Differential Transaction (SDT) method is used to achieve incremental update of drawing and reduce the information redundancy of the blockchain. Finally, a comparative analysis and validation evaluation of the scheme is carried out, and the usability of the scheme is illustrated with an illustrative example. The results show that: (1) proposed scheme is feasible for multi-person collaborative design; (2) proposed scheme can effectively ensure the reliability of drawing and reduce the redundancy of blockchain information, so as to achieve copyright protection for designers.

## Introduction

Currently, Building Information Modeling (BIM) is gradually being used in the design, construction, and maintenance of large-scale buildings^1–5^. Due to the complexity of large-scale construction projects, multiple disciplines such as architecture, structure, and equipment are usually involved in the design, and the workload is large. In order to improve efficiency, BIM multi-person collaborative design is currently mainly based on cloud platforms^6,7^ or the collaborative design function of modeling software^8^. Cloud-based BIM collaborative design (e.g., Autodesk BIM 360, BIMServer) uses the cloud to store and manage BIM drawing data. Although cloud storage has the advantages of high flexibility and strong scalability, due to the influence of centralized management, data security problems such as data loss, tampering and sensitive data leakage are prone to occur^9,10^. The collaborative design function of BIM modeling software such as Revit has higher requirements on network speed and hardware performance^8^, and it is difficult to effectively record the copyright information of BIM drawing and protect the copyright interests of BIM designers. In addition, both of these methods need to be based on trusted third-party services to realize the storage and update of BIM drawing collaborative design results, which is difficult to ensure the safety and reliability of multi-person collaborative design data.

Blockchain^11^ is a distributed ledger with features such as decentralization, traceability, and tamper resistance. It has been successfully applied to IoT systems^12^, Industry 4.0^13^, and electronic medical records^14^. Due to its characteristics of secure data storage and reliable sharing, blockchain technology has been gradually applied to BIM drawing design^15–18^. Reference^19^ proposed an innovative construction project management model that integrated BIM and blockchain to provide new ideas for digital construction and collaborative management of construction projects throughout their life cycle. Kasten^20^ systematically reviewed and summarized the relevant literature on the application of blockchain in BIM and put forward suggestions for further research directions. All the above studies mentioned the application of blockchain to multi-person collaborative design of BIM drawing. Although these studies did not discuss the design or process of related schemes in detail, they provide directions and goals for subsequent research. Reference^21^ used blockchain to record operation information such as uploading, downloading and deleting of BIM drawing in rail transit projects, which ensured the flow of files and the traceability of responsibilities between multiple parties. Tao et al.^22^ used BIM Collaboration Format (BCF) to complete multi-person collaborative design operations on BIM drawing, and used blockchain and InterPlanetary File System (IPFS) to store BCF file generation information and corresponding BCF files, which improved the security and reliability of design data. Shen et al.^23^ proposed a multi-person collaborative creation system for BIM drawing based on blockchain, which used blockchain to record the hash value of BIM drawing design information and realized the integrated storage of BIM drawing. However, these methods mainly record the operation log or the hash value of design information in the design phase, and cannot provide effective copyright proof without recording the specific design content accurately. At the same time, with the advancement of design process, the problem of information redundancy in blockchain has gradually emerged. To solve the above problem, Das et al.^24^ proposed using blockchain to record BIM design information in the trustless environment of construction projects. Xue et al.^25^ proposed a semantic difference transaction (SDT) method for incremental update of drawing, which incrementally updated by calculating the semantic difference of BIM drawing to reduce information redundancy. However, the above methods have not effectively integrated blockchain and BIM drawing in the process of multi-person collaborative design of BIM drawing. Problems such as the consistency of BIM drawing versions, the management of safety and reliability have not been resolved.

In response to the above problems, this paper studies the multi-person collaborative design of BIM drawing based on blockchain, and the main contributions are as follows:

- A multi-person collaborative design model of BIM drawing based on blockchain is proposed. Besides, prototype system is implemented according to the proposed model, which can provide a complete function for multi-person collaborative design of BIM drawing.
- The collaborative storage method based on blockchain (on-chain) and IPFS (off-chain) is adopted, which solves the problem that blockchain cannot store large data such as BIM drawing file. To protect the copyright interests of designers, IPFS is used to ensure the integrity and reliability of BIM drawing.
- A cycle division mechanism is designed to solve the problem of information synchronization during multi-person collaborative design of BIM drawing, which can ensure the consistency of BIM drawing.
- Incremental update of BIM drawing is realized using SDT method, which reduces the information redundancy of BIM.
- The developed system is thoroughly tested through the following aspects: time-consuming of BIM drawing collaborative design, transaction throughput, and BIM drawing consistency. In addition, security and reliability of developed system are also analyzed. Finally, the usability of proposed scheme is verified by an example.

The rest of this paper is arranged as follows: section “Related work” summarizes the literature on integrated application of BIM, blockchain and architecture. Section “Multi-person collaborative design model of BIM drawing” is to propose a multi-person collaborative design model of BIM drawing based on blockchain. Section “Detailed structure and process design of BIM drawing multi-person collaborative design model” is to expound the detailed structure of proposed model and related processes. Section “Experimental verification and evaluation analysis” implements the prototype system, and designs experiments to test and evaluate the relevant indicators and functional processes of this system. Finally, section “Conclusion” concludes the whole paper.

## Related work

### Traditional BIM drawing multi-person collaborative design

Currently, BIM multi-person collaborative design tools or platforms used in large-scale construction projects are mainly divided into two categories: (1) single tools that promote data exchangeability and interoperability, and (2) integrated BIM-based collaborative design platforms that facilitate comprehensive collaboration^22^. The first category of tools facilitates collaborative design by enhancing a specific aspect of BIM data, such as recording BIM design changes^26^, enhancing compatibility of BIM data formats^27^, or version management of BIM data^28^. However, these studies have problems such as low data access and sharing efficiency, and large application limitations. In contrast, the second type of multi-person collaborative design platform does not have the above problems, and it can provide more abundant and practical functions for BIM multi-person collaborative design. Moayeri et al.^29^ proposed a new visualization model, which visualized the changes and modifications of BIM in construction projects. El-Diraby et al.^30^ used related plugins to combine the BIM online design platform with energy simulation software, and ultimately helped people make decisions about “green buildings” through comprehensively considering design and simulation results. Poerschke et al.^31^ conducted research on BIM data collection, analysis and information collaboration. They proposed the methods design collaboration and design presentation between different disciplines in the stage of architectural design. Singh et al.^32^ studied the multi-professional collaboration in the U.S. construction industry, analyzed the problems during the process of data interaction between different disciplines, and proposed a collaboration platform based on BIM server according to the relevant analysis conclusions. Liu et al.^33^ introduced a cloud-based BIM data center, which provided functions such as storage, sharing, and retrieval of BIM-related data. Logothetis et al.^34^ proposed a cloud-based open-source system for storing, viewing and analyzing BIM-related data online.

The tradition studies often store BIM data in the cloud or database for sharing, so as to assist the real-time collaborative design of BIM. These studies improve the understanding of design information in the BIM multi-person collaborative design process and provide practical solutions to facilitate multi-person collaborative design. However, there are still some problems in these schemes, such as the inability to guarantee the security of collaborative data, the confusion of BIM drawing version management, and the redundancy of information, which need further research and improvement.

### Blockchain in construction

Blockchain technology enables highly reliable and traceable data management based on distributed ledgers and consensus mechanisms^35^. Each block in the blockchain has two hash values, “current block hash” and “previous block hash”. The latter block is linked to the previous block by “previous block hash”, and finally a chain is formed. Any slight modification to block information will cause the block hash to change. Therefore, if someone tries to tamper with a block in their local blockchain ledger, the block will be recognized by other blockchain nodes because of the hash mismatch^36^. Not only that, no single blockchain node can manipulate all the data in the blockchain. Because only when a malicious blockchain node holds more than 50% of the computing power, it is possible to control the entire blockchain network, which is almost impossible from the current technical point of view^18,37^. Therefore, blockchain can guarantee the security and trustworthiness of data by keeping the same, unalterable and traceable copy of complete data in each node^38^.

In recent years, the application of blockchain in the construction field has been increasing, and it has played an important role in driving the digital transformation in construction field, reducing costs^39^, accelerating collaboration and maximizing trust^40^. For example, Wen et al.^41^ proposed a blockchain-enhanced price-incentivized demand response framework for demand-side management, enabling optimal energy scheduling among multiple users within a building. Van et al.^42^ proposed a blockchain-based distributed cooperative energy response framework for managing the use of renewable energy by users in buildings. Das et al.^43^ proposed a distributed building construction document management system based on blockchain and distributed content addressable storage, which promoted the smooth flow of construction information among different project participants and improved the quality of construction projects. Wang et al.^44^ proposed a blockchain-based framework for enhancing supply chain traceability in off-site construction. Lee et al.^45^ proposed a framework combining blockchain and digital twins to track data records in construction projects. Das et al.^46^ developed a blockchain-based construction project payment framework, utilized smart contracts to automate payments and share payment records among all members. Li et al.^47^ applied blockchain to the buildings internet of things system to improve the sustainability of construction projects and the reusability of data. Sheng et al.^48^ designed a construction quality chain based on blockchain to ensure the security of construction quality information and enhance the trust among construction project members. Zheng et al.^18^ used the blockchain to record the whole process design information of BIM, which ensured the security and traceability of design information. Liu et al.^16^ explored the potential role of the integration of BIM and blockchain for sustainable building design information management, and used blockchain to assist BIM for sustainable building design coordination and collaboration across multiple construction stages. Lokshina et al.^49^ studied the integrated application of blockchain, BIM and buildings internet of things system. They proposed that the integrated application of these three can provide an innovative framework for digital transformation of construction industry. Nawari et al.^50^ investigated the potential integration of blockchain and BIM processes by conducting a survey of blockchain and its application in a built environment. Li et al.^51^ focused on digital transformation of construction industry, reviewed current technology, environment, conceptual models, and discussed the application and development trend of blockchain technology in digital construction.

In summary, blockchain has attracted extensive attention in the construction field due to its potential in many aspects such as data security, data traceability, identity authentication, access control, and traceability management of BIM and supply chains. Blockchain can record and save the complete modification and change history of BIM, which has great potential in the field of building safer digital buildings^39,51^. Therefore, safe, precise and efficient management during the digital process of construction projects depend on the effective integration and support of blockchain and BIM^52^.

### Multi-person collaborative design of BIM drawing based on blockchain

In view of the situation that large-scale construction projects often involve multiple parties, blockchain is gradually used to solve the problems of multi-party trust and security traceability in BIM design. Winfield^38^ pointed out in the report that blockchain can provide synchronized, tamper-proof and traceable data records, showing great potential to solve the problem of BIM collaborative design. Elghaish et al.^40^ asserted that the organic integration of blockchain and BIM can easily track all BIM changes in design and construction phases of the building project. Nawari et al.^53^ believed that blockchain can play an advantage in BIM collaborative workflow by providing secure and reliable data storage. Li et al.^54^ used blockchain to protect intellectual property, data ownership and copyright in BIM collaborative design. Zheng et al.^18^ proposed using blockchain to record the changes of BIM drawing during the collaborative design process, thereby ensuring the credibility and security of the design process. Most of the current researches combining blockchain and BIM multi-person collaborative design are conceptual frameworks^55^, which are given and compared in Table 1.

**Table 1:** Research on the combination of blockchain and BIM multi-person collaborative design.

References	Data storage	On-chain data	Off-chain storage	Consolidated storage	Consistency	Information redundancy
Wang et al. (described in this article)	Mixed	BIM design content	IPFS	Yes	Yes	No
Duan et al.^21^	Blockchain	BIM complete information and design operation information	/	No	No	Yes
Tao et al.^22^	Mixed	summary of BIM design operation information	IPFS	No	No	Yes
Shen et al.^23^	Mixed	summary of BIM complete information	Database	Yes	Yes	Yes
Das et al.^24^	Mixed	BIM design content	Database	No	No	Yes
Xue et al.^25^	Blockchain	BIM design content	/	No	No	No

From the perspective of data storage methods, there are mainly two methods: blockchain storage and hybrid storage. Among them, blockchain storage can ensure the safety and reliability of data on the chain. However, due to block size and other reasons, blockchain is not suitable for storing large files such as BIM drawing and cannot protect BIM drawing files and BIM drawing change information at the same time. Literature^21^ tried to use blockchain to store the complete information of BIM drawing, but it would bring serious information redundancy problems^25^. Compared with blockchain storage, hybrid storage is more flexible and efficient. The basic mode is to use blockchain to store brief abstracts or design information, and to store BIM drawing files in off-chain. The difference lies in the choice of off-chain storage methods. Shen et al.^23^ and Das et al.^24^ used traditional databases as off-chain storage methods, which faces security risks such as data tampering and leakage^56^. In response to this problem, literature^22^ introduced IPFS, a new distributed file storage protocol, to ensure the authenticity and credibility of stored files through operations such as partitioning and encryption. It has great potential to solve the problem of inefficient and huge data storage in blockchain^57,58^.

From the perspective of data storage content in blockchain (on-chain), literatures^21,22^ and^23^ used blockchain to store the complete information of BIM drawing or the summary of BIM drawing design operation records, which has encountered two problems. On the one hand, the complete information of BIM drawing is relatively large, and a large part of it is design-independent which does not need to be stored. Using blockchain to store it will take up lots of storage space. In addition, due to data synchronization between blockchain nodes, information redundancy in blockchain is further increased. On the other hand, the summary of BIM drawing design operation information cannot provide accurate design content, so it cannot be used as a valid copyright proof. In contrast, literature^24^ utilized blockchain to store accurate design content, and literature^25^ further proposed the SDT method to minimize the design content that needs to be stored and reduces information redundancy in blockchain.

From the perspective of design process, two issues are mainly considered: (1) how to deal with conflicts between BIM drawing versions and (2) how to ensure the consistency of BIM drawing. As shown in Table 1, only the literature^23^ considered these two issues. It added version number information for each design and implemented merged storage according to the version number when a conflict occurred. Besides, all designers were required to re-obtain the latest BIM drawing at the beginning of each round of design to ensure the ultimate consistency of BIM drawing. However, there are still some problems such as low efficiency and large storage space needed to be solved in this scheme.

This section compares and analyzes the existing researches on the combination of blockchain and BIM multi-person collaborative design from the perspectives of data storage methods, data storage content in blockchain, and design processes. It can be seen from the analysis results that each scheme has advantages and disadvantages, and there are still some problems to be solved urgently. This paper will give feasible solutions to these problems.

## Multi-person collaborative design model of BIM drawing

Aiming at the current issues of security, consistency, redundancy and copyright in the multi-person collaborative design of BIM drawing, this paper studies the multi-person collaborative design of BIM drawing based on blockchain, and mainly solves the following problems in the traditional multi-person collaborative design of BIM drawing:

1. Aiming at the problem of data security and lack of trust, the on-chain and off-chain collaborative storage method of blockchain and IPFS is adopted. Mutual trust and supervision among BIM drawing designers are achieved through the consensus mechanism to improve data reliability. Meanwhile, the security of the data can be ensured through the anti-tampering feature of blockchain.
2. Aiming at the problem of information fusion and consistency, the cycle division mechanism is used to avoid the problem of information synchronization during design and ensure the consistency of BIM drawing among designers.
3. For the problem of BIM drawing information redundancy and copyright, the SDT method is used to achieve incremental updates of BIM drawing, and the blockchain information records are used to provide reliable copyright proofs to safeguard the designer's copyright interests.

In order to facilitate the following discussion, unified data description is defined in Table 2.

**Table 2:** Formal description of stored content.

Symbol	Description
$Addr_{IPFS}$	Hash storage address of BIM drawing in IPFS
$D_{i}$	BIM drawing, $D_{init}$, $D_{new}$, $D_{update}$ respectively indicate the initial, newest, and updated BIM drawing
$DAI_{record}$	BIM drawing summary record
$DAI\_C_{j}$	BIM drawing summary related smart contracts, $DAI\_C_{upload}$, $DAI\_C_{download}$ respectively indicate BIM drawing summary upload and download smart contract
$DDI$	BIM drawing design information
$DDI_{record}$	BIM drawing design information record
$DDI\_C_{k}$	BIM drawing design information related smart contracts, $DDI\_C_{upload}$, $DDI\_C_{download}$ respectively indicate BIM drawing design information upload and download smart contract
$DFDI$	BIM drawing fusion design information
$DFDI_{record}$	BIM drawing fusion design information record
$DFDI\_C_{m}$	The merged BIM drawing design information related smart contracts, $DFDI\_C_{upload}$, $DFDI\_C_{download}$ respectively indicate the merged BIM drawing design information upload and download smart contract
$H_{n}$	Hash value, $H_{D}$, $H_{DDI}$, $H_{DFDI}$ respectively indicate the hash value of BIM drawing content, $DDI$ and $DFDI$
$Sig_{p}$	Digital signature, $Sig_{H\_D}$, $Sig_{H\_DDI}$ respectively indicate the digital signature of $H_{D}$ and $H_{DDI}$

### Logical framework

**Figure 1:**
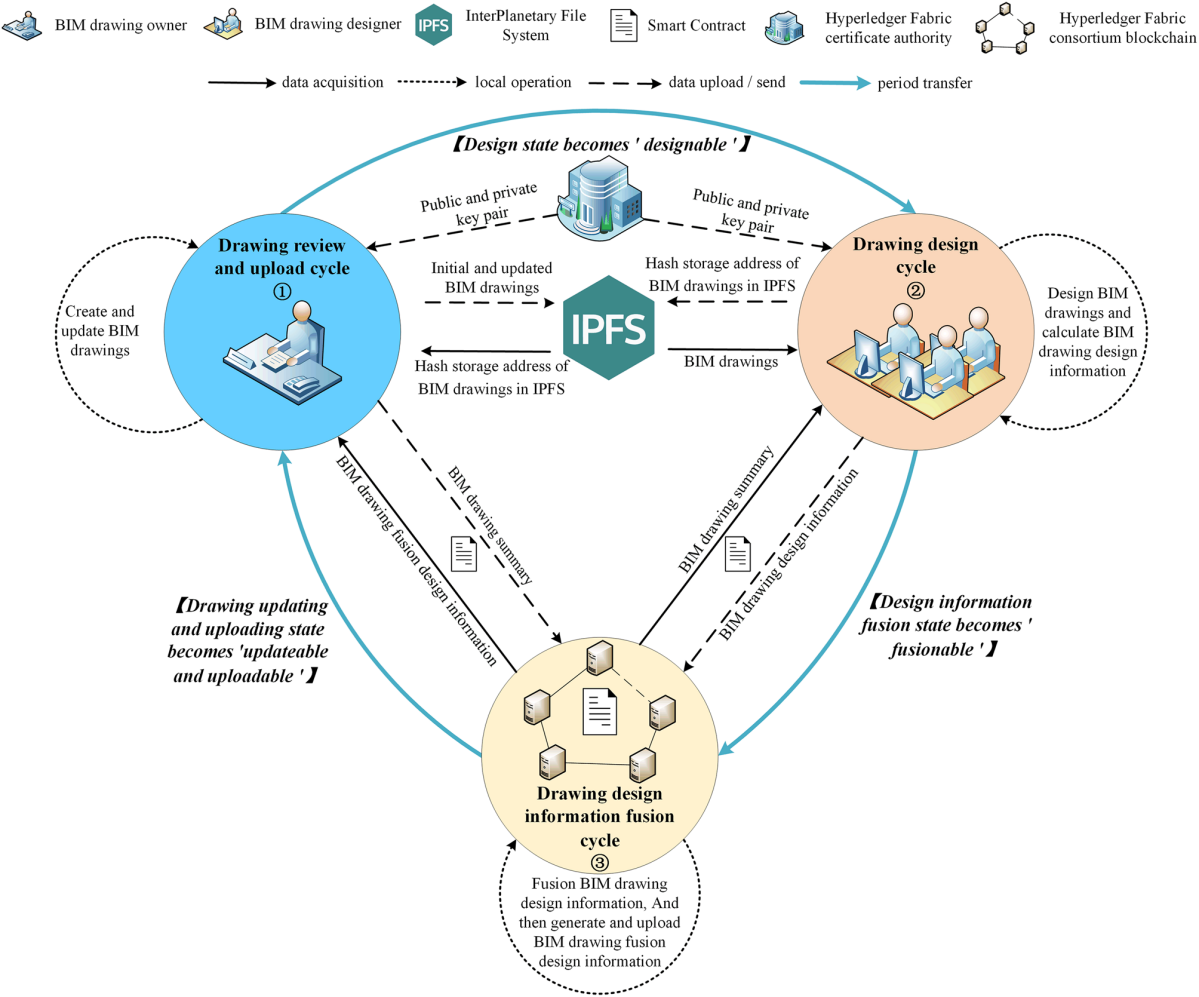
The logical framework of multi-person collaborative design of BIM drawing based on blockchain.

The logical framework of multi-person collaborative design of BIM drawing based on blockchain is shown in Fig. 1. The model is implemented based on Hyperledger Fabric due to the excellent performance and high security which can meet the needs of actual scenarios. The main roles in this model include: Hyperledger Fabric certificate authority (CA), Hyperledger Fabric consortium blockchain (BC), InterPlanetary File System (IPFS), BIM drawing owner (BIM_O) and BIM drawing designer (BIM_D).

The specific tasks of each role are shown in Table 3:

**Table 3:** The specific tasks of each role within the logical framework.

Role	Specific tasks
CA	Generating public/private key pairs for BIM_O and BIM_D
BC	Integrating $DDI$ to generate $DFDI$, storing data (e.g.,$DDI_{record}$, $DFDI_{record}$, $DAI_{record}$, etc.) and performing data encryption, signature and verification
IPFS	Storing complete BIM drawing
BIM_O	Updating and uploading BIM drawing according to $DFDI$
BIM_D	Acquiring and designing BIM drawing, then uploading them after calculating $DDI$

### Cycle division mechanism

**Figure 2:**
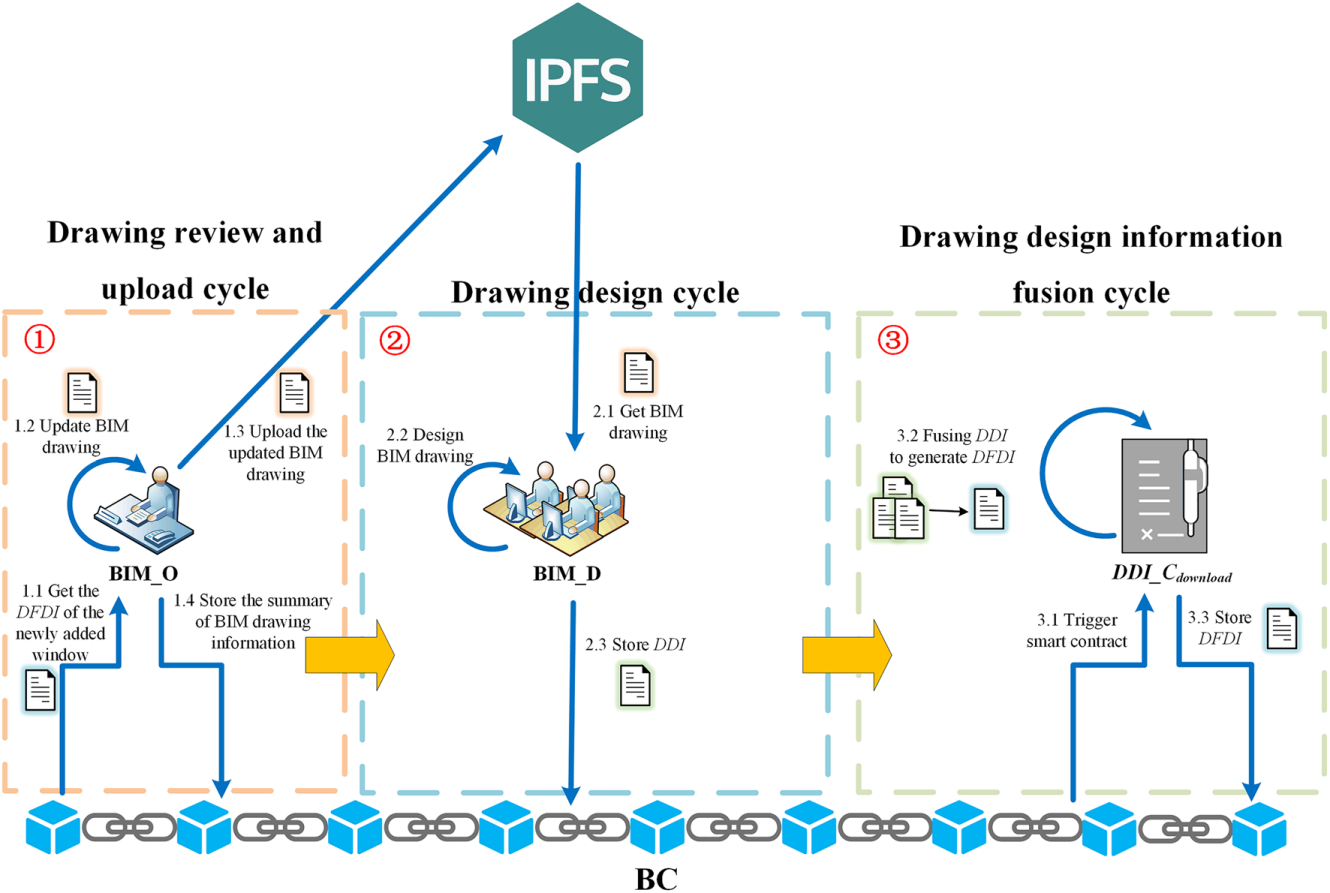
Example of cycle division and circulation.

During the multi-person collaborative design of BIM drawing, in order to ensure the information synchronization among designers and improve the design efficiency, the entire process of multi-person collaborative design is divided into three cycles: drawing review and upload cycle, drawing design cycle and drawing design information fusion cycle. As shown in Fig. 2, the cycle division process is illustrated by adding a window to BIM drawing as an example. A specific role performs the specific operation in each cycle, and each operation is not performed across cycles. The specific divisions are as follows:

1. Drawing review and upload cycle: BIM_O performs operations, including BIM drawing update, BIM drawing upload and $DFDI$ acquisition operation implemented through smart contract.
2. Drawing design cycle: BIM_D performs operations, including BIM drawing acquisition, BIM drawing design and $DDI$ calculation and upload operation implemented through smart contract.
3. Drawing design information fusion cycle: BC automatically calls smart contract to execute operations of $DDI$ fusion and upload after each BIM_D uploads $DDI$ to BC.

Each cycle is independent, and conversion between cycles is achieved through the change of system status bit. When the drawing status is ‘updateable and uploadable’, enter the drawing review and upload cycle; when the status is ‘designable’, enter the drawing design cycle; when the status is ‘fusionable’, enter the drawing design information fusion cycle. Based on cycle division mechanism, all BIM_D obtain the latest BIM drawing uploaded by BIM_O from the IPFS at the beginning of the drawing design cycle, and then design the BIM drawing. This enables BIM_D to design on the same latest BIM drawing, which ensures the information synchronization when multiple people are designing.

### Information storage

**Figure 3:**
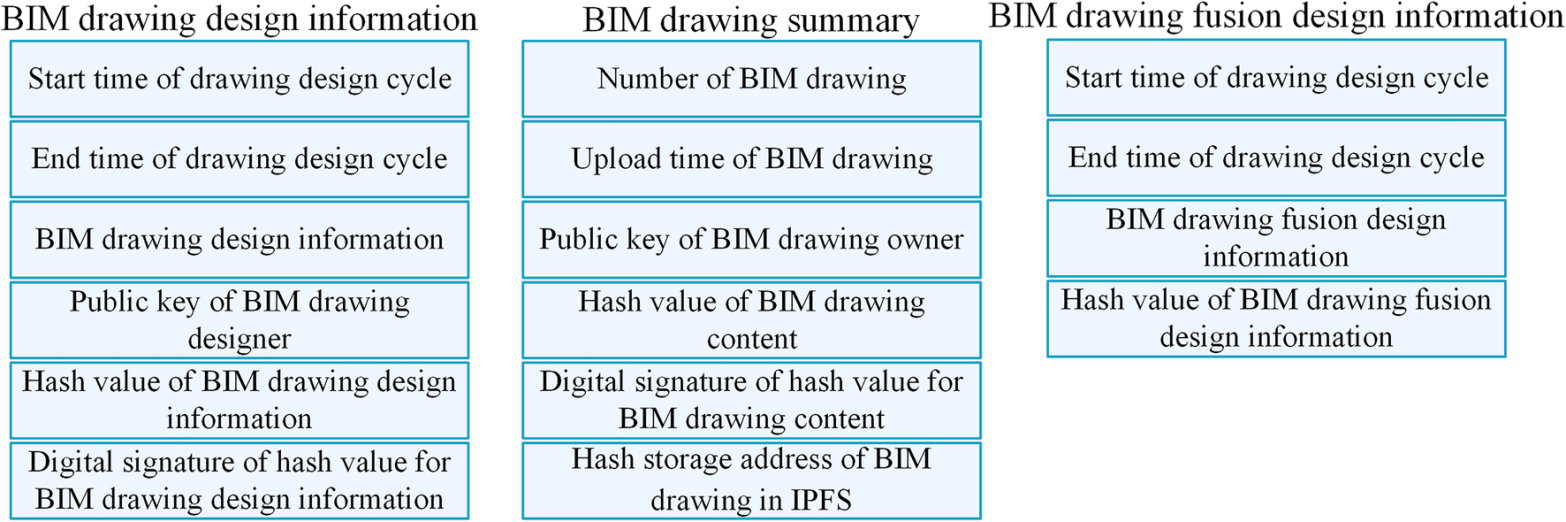
On-chain storage of BC.

This paper combines BC and IPFS to implement the on-chain (BC) plus off-chain (IPFS) hybrid storage of data. Off-chain (IPFS) stores BIM drawing, while the main content stored in BC is shown in Fig. 3.

**Figure 4:**
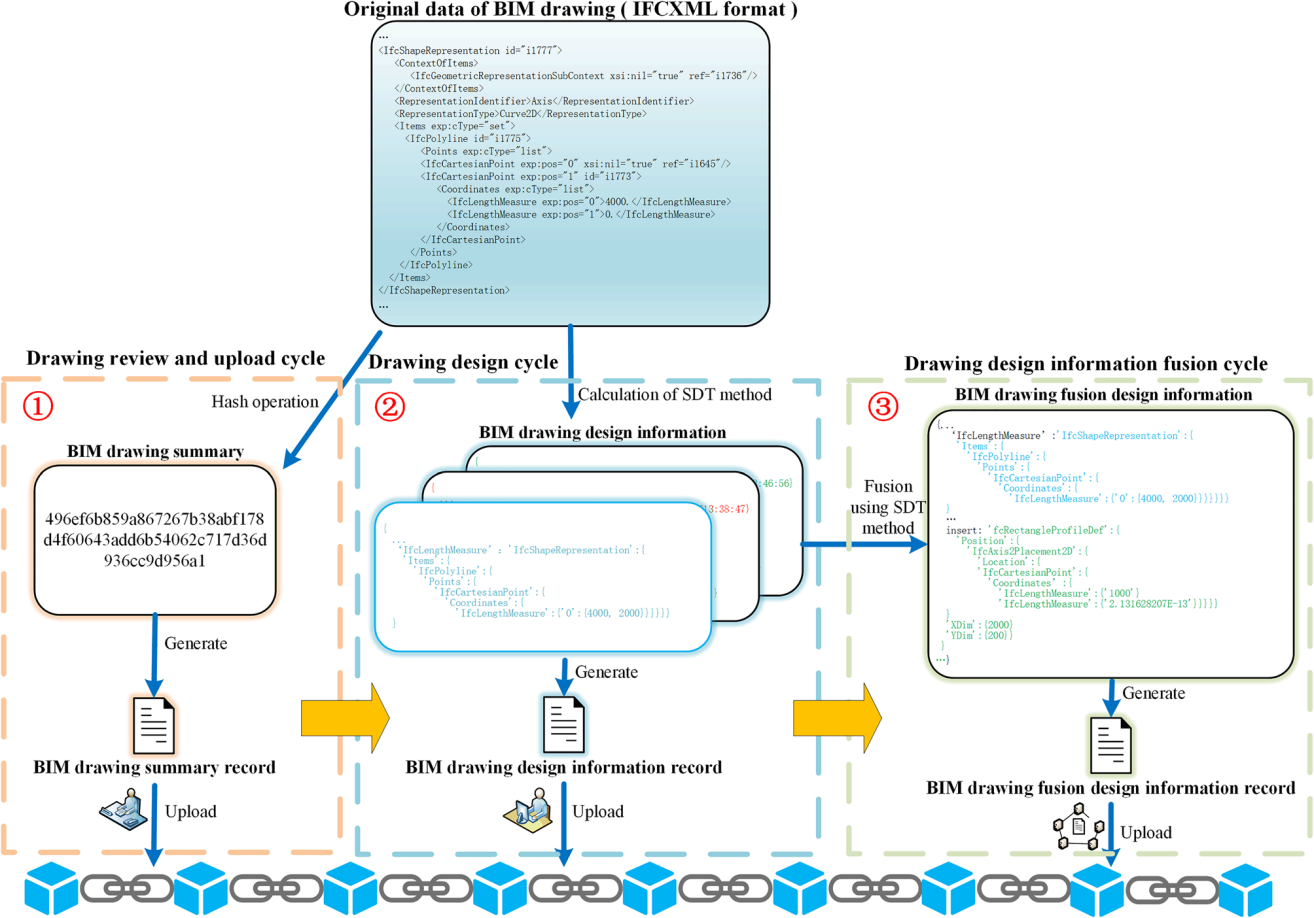
An example of BIM data storage content changes on BC.

Different roles upload different BIM data to blockchain in different cycles. An example of BIM data storage content changes on the BC is shown in Fig. 4. Three types of data are uploaded to BC by three roles in three cycles: (1) drawing review and upload cycle, the original data of BIM drawing file is obtained through hash operation by BIM_O, and the BIM drawing summary is generated and uploaded to BC; (2) drawing design cycle, BIM_D uses SDT method to calculate the original data of BIM drawing file to obtain $DDI$, generates $DDI_{record}$ and uploads it to BC; (3) drawing design information fusion cycle, smart contract deployed in BC automatically uses SDT method to fuse each $DDI$ to obtain $DFDI$, generates $DFDI_{record}$ and uploads it to BC.

### Running process

**Figure 5:**
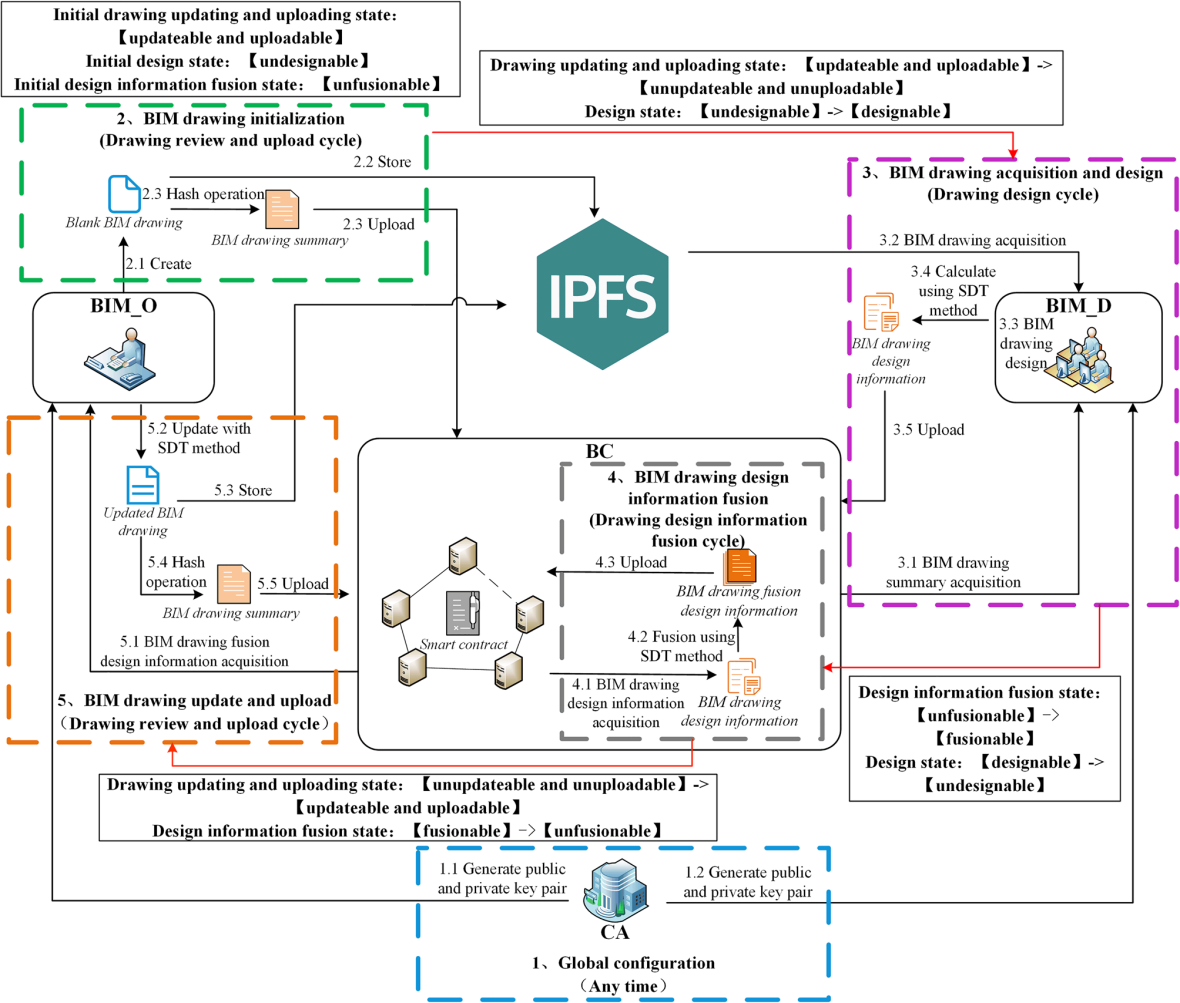
Blockchain-based BIM drawing multi-person collaborative design model running process.

As shown in Fig. 5, the running process of model includes the following steps:

**Step 1:** Global configuration. It is executed by CA. The public and private key pairs are automatically generated when a new BIM_O or BIM_D is added to the model.

**Step 2:** BIM drawing initialization. It belongs to drawing review and upload cycle. BIM_O creates $D_{init}$ and stored in IPFS, and the returned $Addr_{IPFS}$ is obtained. $H_{D}$ is calculated according to $D_{init}$ and then passes $H_{D}$、$Addr_{IPFS}$ to $DAI\_C_{upload}$. $DAI\_C_{upload}$ calculates $Sig_{H\_D}$ and combines other relevant information to generate $DAI_{record}$, then uploads $DAI_{record}$ to BC.

**Step 3:** BIM drawing acquisition and design. It belongs to the drawing design cycle. BIM_D obtains the latest BIM drawing for design. BIM_D first uses $DAI\_C_{download}$ to acquire $DAI_{record}$ from BC, and then verifies whether $H_{D}$ has been tampered. If not, BIM_D obtains $D_{new}$ from the IPFS according to the $Addr_{IPFS}$, verifies the correctness of $D_{new}$, then designs on $D_{new}$ that has not been tampered.

Before the end of the drawing design cycle, BIM_D uses SDT method to obtain $DDI$ and calculate $H_{DDI}$, and then $H_{DDI}$ is transferred to $DDI\_C_{upload}$. $DDI\_C_{upload}$ calculates $Sig_{H\_DDI}$, combines other relevant information to generate $DDI_{record}$ and uploads it to BC.

**Step 4:** BIM drawing design information fusion. It belongs to the drawing design information fusion cycle. BC automatically uses $DDI\_C_{download}$ to obtain each $DDI_{record}$ and verifies whether $H_{DDI}$ and $DDI$ have been tampered. If not, SDT method is used to merge all $DDI$ into $DFDI$. $DDI\_C_{download}$ calculates $H_{DFDI}$ and combines other relevant information to generate $DFDI_{record}$. Finally, $DDI\_C_{download}$ calls $DFDI\_C_{upload}$ which uploads $DFDI_{record}$ to BC.

**Step 5:** BIM drawing update and upload. It belongs to the drawing review and upload cycle. BIM_O uses $DFDI\_C_{download}$ to obtain $DFDI_{record}$ from the BC, and then verifies whether $DFDI$ in it has been tampered. If not, SDT method is used to update local BIM drawing of BIM_O according to $DFDI$, then store $D_{update}$ to IPFS. Finally, generate $DAI_{record}$ and upload it into BC.

## Detailed structure and process design of BIM drawing multi-person collaborative design model

Corresponding to the model designed in the previous section, this section introduces the detailed structure and related processes of the model.

### Global configuration

**Figure 6:**
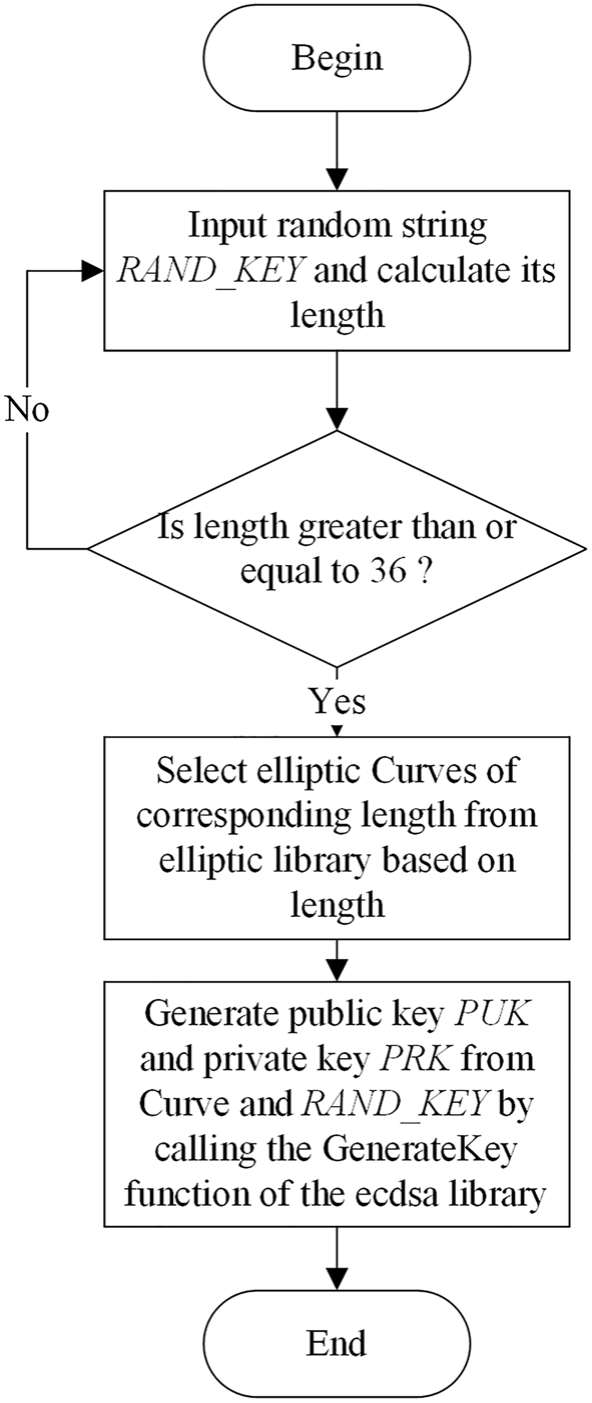
Public and private key pair generation process.

When new BIM_O or BIM_D join the model, CA automatically generates public/private key pair for him/her. As shown in Fig. 6, $RAND\_KEY$ needs to re-input when its length is less than 36.

### BIM drawing initialization

**Figure 7:**
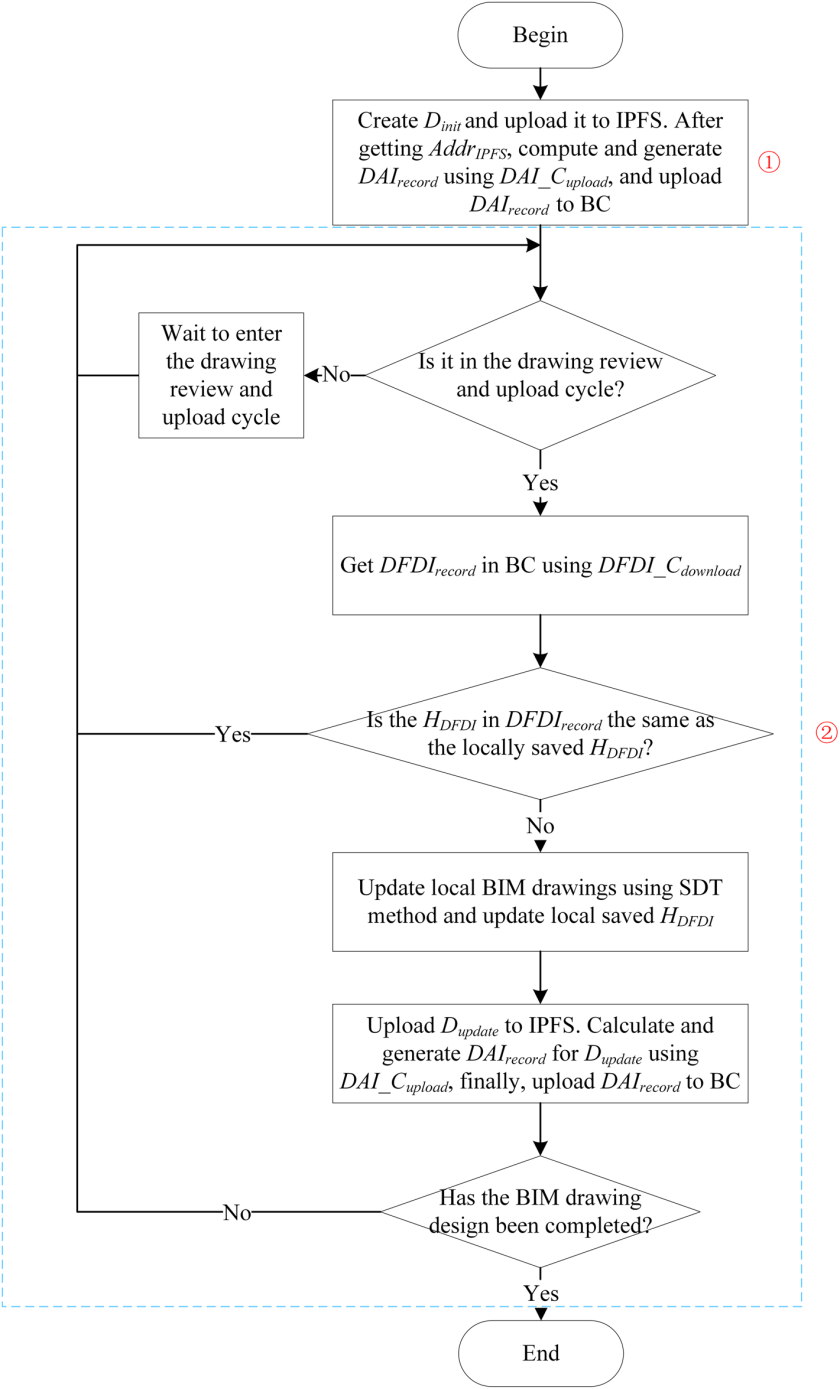
Operation process of BIM drawing owner.

The multi-person collaborative design process of BIM drawing starts with the drawing review and upload cycle. In the first drawing review and upload cycle, BIM_O creates $D_{init}$ for drawing initialization. In order to explain the operation of BIM_O during this cycle, the complete operation process of BIM_O is first shown in Fig. 7. The initial steps of BIM drawing correspond to serial number ➀, and serial number ➁ corresponds to the steps of BIM drawing updating and uploading, which will be introduced in detail in section “BIM drawing update and upload”.

After BIM_O creates $D_{init}$ and uploads it to IPFS, $DAI\_C_{upload}$ is called to calculate and generate $DAI_{record}$ according to the related information of $D_{init}$. $DAI_{record}$ includes the number of BIM drawing, upload time of BIM drawing, public key of BIM_O, $H_{D}$, $Sig_{H\_D}$ and $Addr_{IPFS}$. $H_{D}$ is calculated by $SHA256()$ hash function, and $Sig_{H\_D}$ is generated by using the private key of BIM_O. $Addr_{IPFS}$ is obtained by hashing $D_{init}$ and Base58 encoding inside IPFS.

### BIM drawing acquisition and design

**Figure 8:**
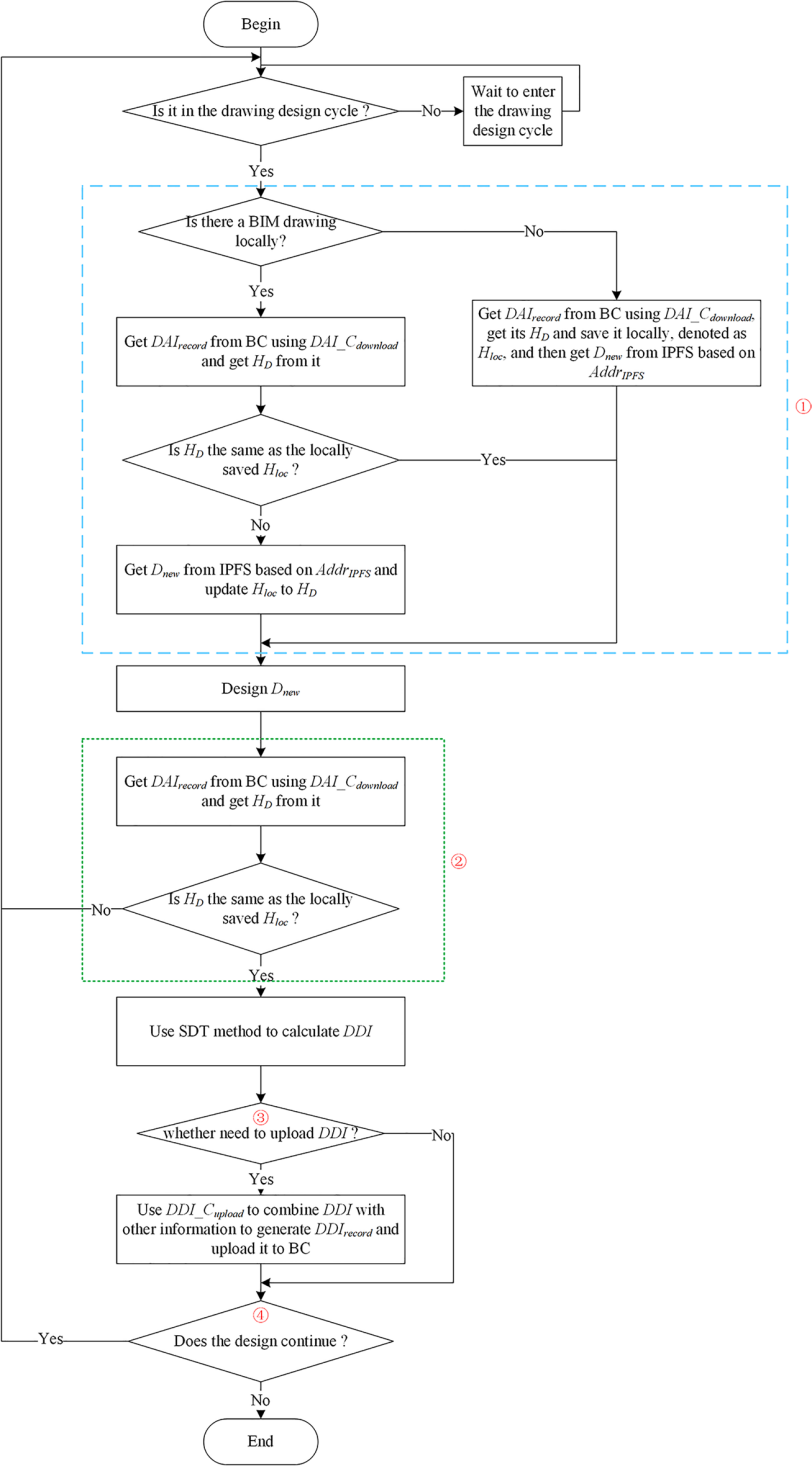
The operation process of BIM drawing designer.

Related operations of BIM drawing acquisition and design are completed by BIM_D within drawing design cycle. The complete process is shown in Fig. 8.

At the beginning of the drawing design cycle, BIM_D obtains $D_{new}$ for design according to the judgment process of number ➀ in Fig. 8 (Assuming BIM_D has learned design requirements of this BIM drawing through other means, $D_{new}$ does not contain any design requirements). Algorithm 1 gives the pseudocode corresponding to the sequence number ➀ processing flow. Firstly, BIM_D obtains $DAI_{record}$ from BC and verifies whether $H_{D}$ has been tampered:

1. BIM_D has BIM drawing locally. If $H_{D}$ has not been tampered, compare $H_{D}$ and $H_{loc}$:
  If equal. It means BIM_D has latest BIM drawing locally, and then BIM_D designs directly.
  If not equal. BIM_D obtains $D_{new}$ from IPFS through $Addr_{IPFS}$ and verifies the correctness of $D_{new}$. If verification passed, BIM_D designs on $D_{new}$, and updates $H_{loc}$ to $H_{D}$.
  If $H_{D}$ is tampered, BIM_D needs to acquire the latest $H_{D}$ from BC which has not been tampered, and obtains BIM drawing from IPFS through $Addr_{IPFS}$ for the next design.

2. BIM_D has no BIM drawing locally. If $H_{D}$ has not been tampered, BIM_D obtains $D_{new}$ from IPFS and verifies the correctness of $D_{new}$, design on $D_{new}$ which has not been tampered, saves $H_{D}$ locally as $H_{loc}$ in order to determine whether $D_{new}$ is the latest BIM drawing. If $H_{D}$ has been tampered, notify BIM_O to regenerate and upload $D_{new}$.

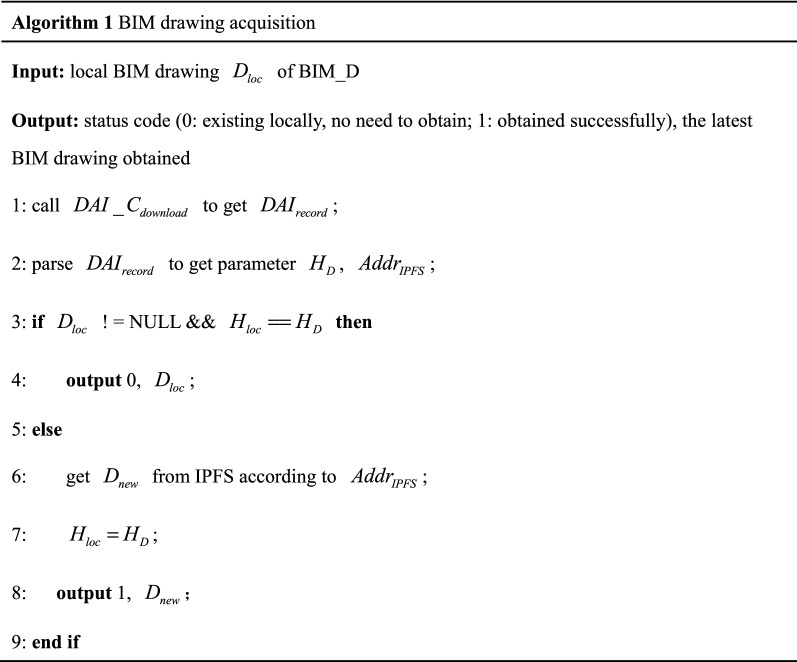


**Figure 9:**
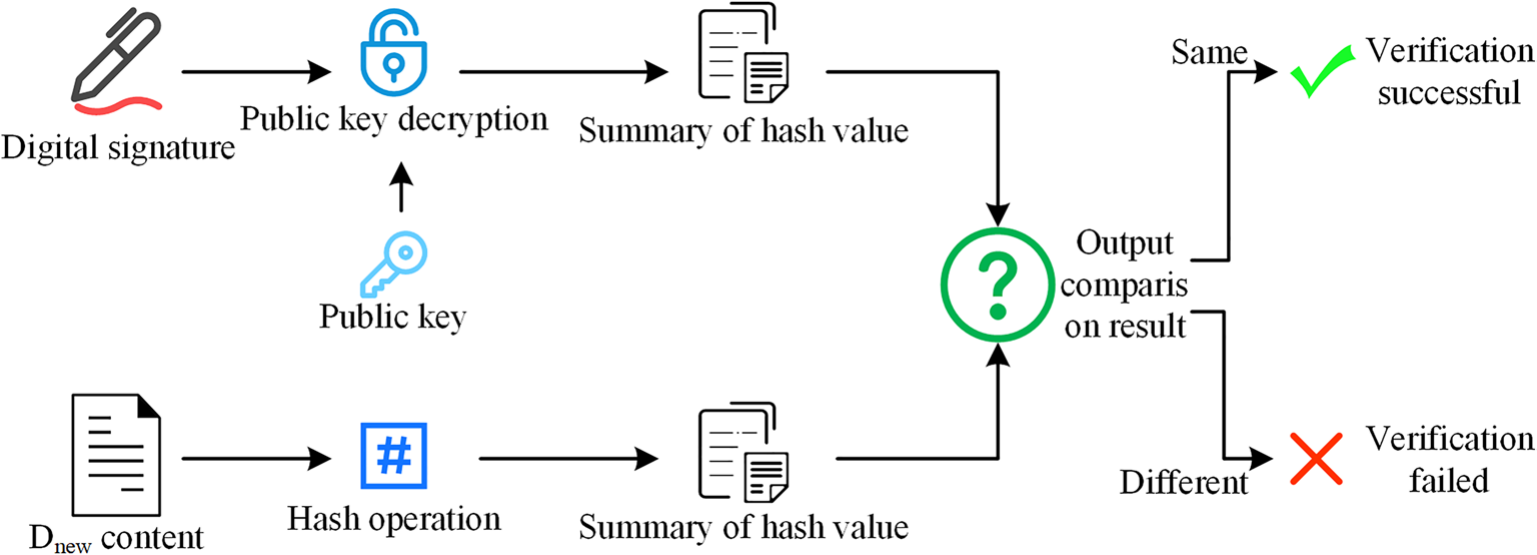
Digital signature verification process.

The above judgment processes involve digital signature verification of $H_{D}$ and $D_{new}$. The verification process is shown in Fig. 9. First, digital signature is decrypted with the public key of corresponding user to obtain encrypted digest. Secondly digest is obtained by re-hashing the original $D_{new}$, and compare whether the two digests are equal. If equal, means $D_{new}$ has not been tampered; otherwise, means $D_{new}$ has been tampered and requires further processing by BIM_O. Each verification process of digital signature in this paper are the same, just replace the digital signature, $D_{new}$, and public key in Fig. 9 to unverified content, its digital signature, and public key of corresponding user.

For some reasons, BIM_D may not calculate $DDI$ in time at the end of the period of drawing design cycle A, and BIM_D may also not reacquire $D_{update}$ at the beginning of next drawing design cycle B. In this case, at the end of drawing design cycle B, the uploaded $DDI$ by BIM_D is actually from cycle A, not in cycle B. It will lead to an error when update BIM drawing in next drawing review and upload cycle during. Considering above possible problem, before BIM_D uses SDT method to calculate $DDI$ in drawing design cycle, it needs to verify whether BIM_D is designed on $D_{new}$, as shown in Fig. 8 ➁.

**Figure 10:**
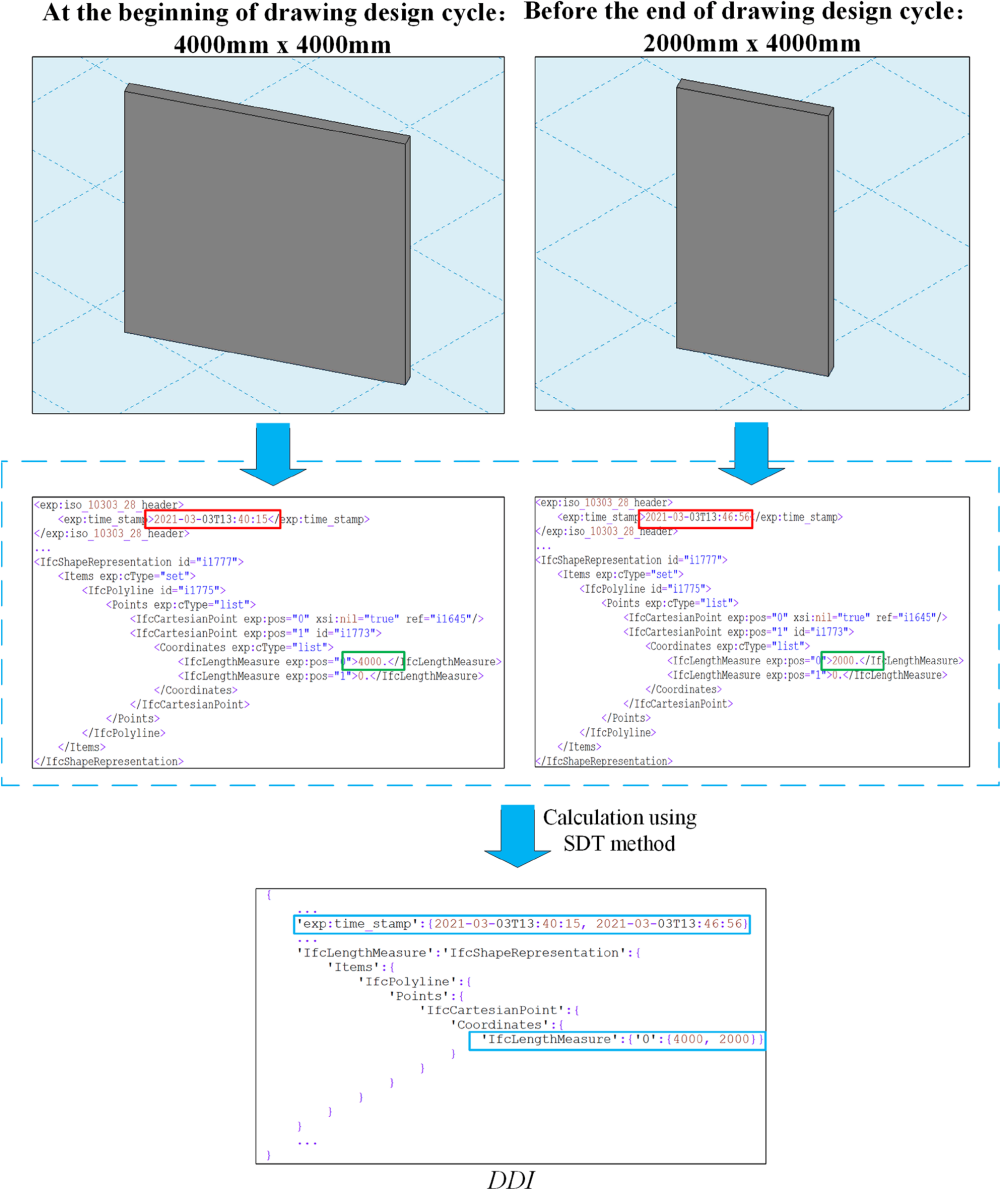
Example of calculating and obtaining BIM drawing design information.

After the above relevant verifications are passed, SDT method is used to calculate $DDI$ in drawing design cycle. BIM drawing is exported to IFC or IFCXML files at the beginning and before the end of drawing design cycle (in this paper, BIM drawings uniformly use IFCXML format). Calculating contents of two files to get $DDI$ in drawing design cycle through jsondiff library of Python. $DDI$ only contains the content difference information of BIM drawing before and after this drawing design cycle, which reduces the information redundancy. As shown in Fig. 10, red and green boxes respectively indicate time and wall length at the beginning and end of drawing design cycle, which show the differences between files before and after the design. The $DDI$ of time and wall length in blue boxes can be calculated through the calculation method of $DDI$.

Then BIM_D decides whether to upload $DDI$ during this drawing design cycle and whether to continue designing BIM drawing. As shown in number ➂ and ➃ in Fig. 8. If BIM_D needs to upload $DDI$, BIM_D will use $DDI\_C_{upload}$ to upload $DDI_{record}$(includes $DDI$, start time of drawing design cycle, end time of drawing design cycle, public key of BIM_D, $H_{DDI}$ and $Sig_{H\_DDI}$) to BC.

### BIM drawing design information fusion

There are large numbers of BIM_D in actual scenarios, and there are many $DDI$ uploaded at the end of drawing design cycle. It will be very cumbersome to obtain these $DDI$ separately to update BIM drawing. In order to facilitate BIM_O to update BIM drawing, $DDI\_C_{download}$ will be automatically executed by BC to merge all $DDI$ into $DFDI$ and upload it to BC after each BIM_D uploads $DDI$ to BC. Therefore, BIM_O only needs to obtain $DFDI$ once to complete the update of BIM drawing.

**Figure 11:**
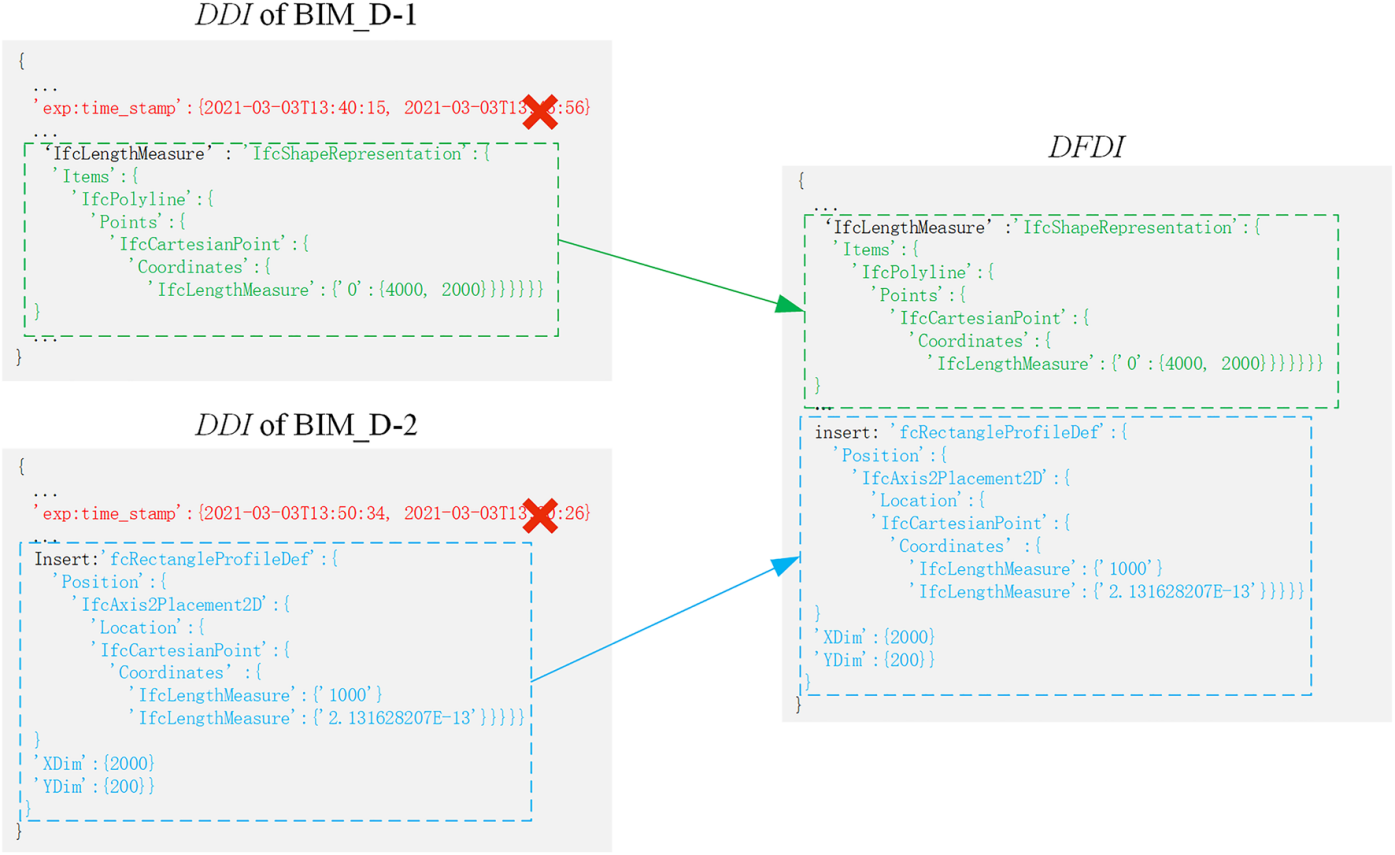
BIM drawing design information fusion example.

In drawing design information fusion cycle, $DDI\_C_{download}$ obtains $DDI_{record}$ and verifies whether $DDI$ in $DDI_{record}$ has been tampered through digital signature in Fig. 9. If validation passed, $DDI$ is merged into $DFDI$ through SDT method. A specific example is shown in Fig. 11. $DDI$ in BIM_D-1 was calculated through SDT method in Fig. 10, as shown in the green font; $DDI$ in BIM_D-2 mainly includes attribute information of newly added window, as shown in the blue font. The $DDI$ fusion rule use the rule in SDT method, as follows: If there is no conflict in $DDI$, just merge directly, otherwise delete all conflicting $DDI$. According to the $DDI$ fusion rule, since $DDI$ in BIM_D-1 and BIM_D-2 both have changes of design date (as shown in red font), delete all the conflicting $DDI$ of date, and then merge the remaining non-conflicting $DDI$ directly to get $DFDI$.

Finally, $DDI\_C_{download}$ combines $DFDI$, start time and end time of drawing design cycle, $H_{DFDI}$ into $DFDI_{record}$ and uploads it to BC through $DFDI\_C_{upload}$ for BIM_O to update BIM drawing in the next step.

### BIM drawing update and upload

When entering the drawing review and upload cycle again, BIM_O will perform operations of BIM drawing update and upload shown in Fig. 7 ➁. Except for the new operation of calling $DFDI\_C_{download}$ to obtain $DFDI$ and using SDT method to update BIM drawing, the other operations are the same as initialization of BIM drawing, so it is not repeated here. We give the pseudocode of the BIM drawing update operation, as shown in Algorithm 2 (corresponding to the processing flow in the loop body of number ➁ in Fig. 7).


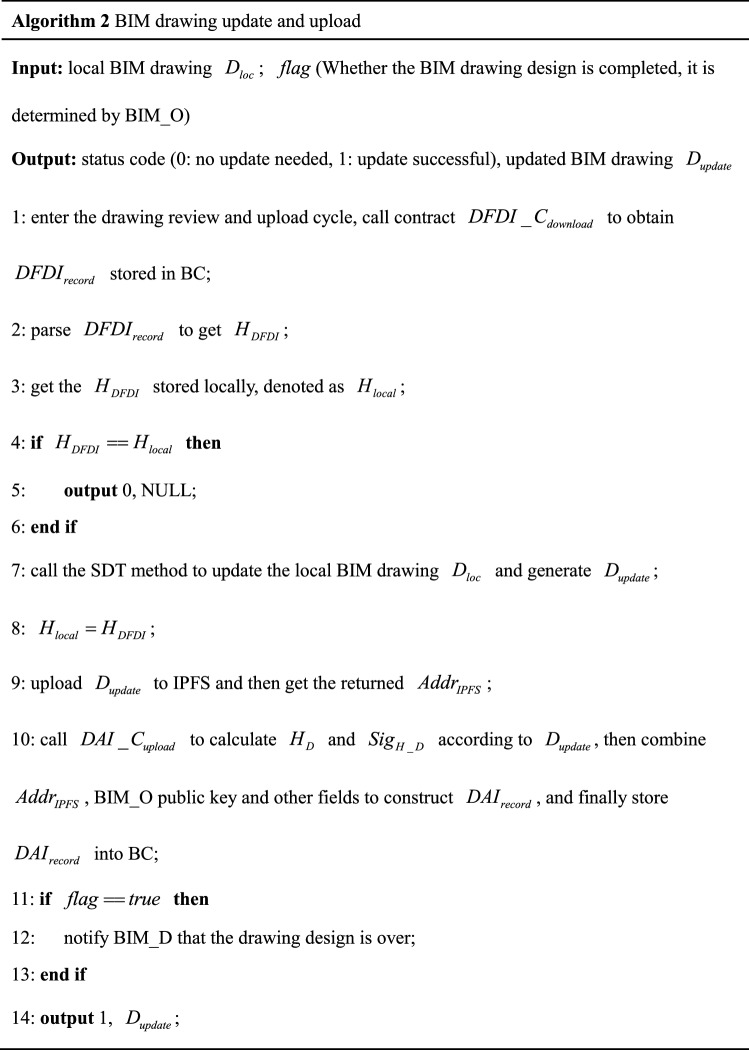


**Figure 12:**
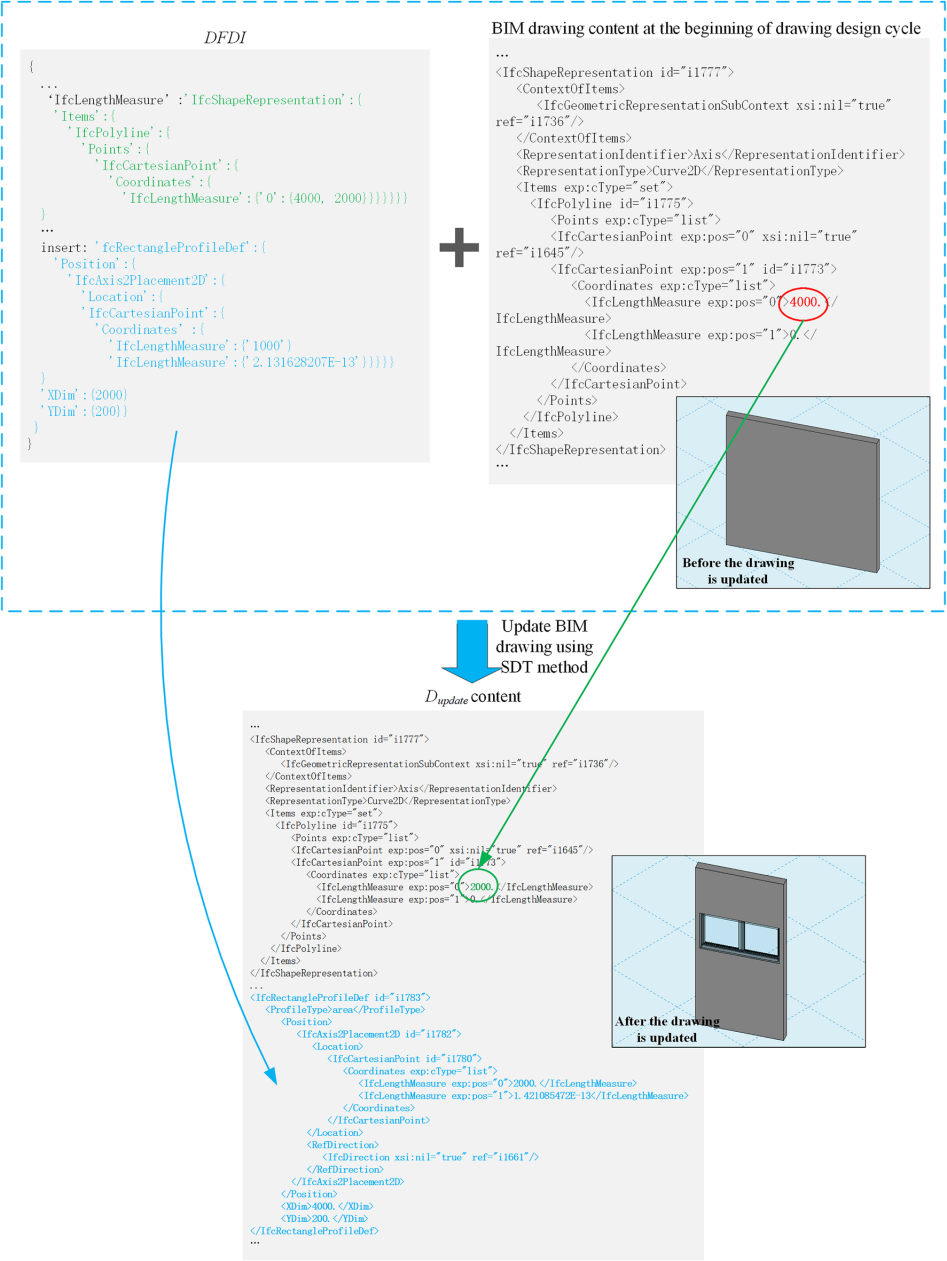
BIM drawing update example.

The SDT method is introduced through a BIM drawing update example. Figure 12 shows an example of BIM drawing update. The $DFDI$ in Fig. 12 obtained from Fig. 11. The green font is the $DDI$ obtained by changing the wall length in BIM_D-1, and the blue font is the $DDI$ obtained by adding a window component to BIM_D-2. $DFDI$ updates BIM drawing method as follows: traverse the hierarchical relationship of component element in $DFDI$, find the component element attribute of the corresponding hierarchical relationship in BIM drawing before update and make corresponding changes. If the corresponding component element attribute is not found, directly add attributes directly to BIM drawing before the update. Therefore, after updating with $DFDI$, the length attribute of wall changed from 4000 mm in BIM drawing at the beginning of drawing design cycle to 2000 mm in $D_{update}$, the relevant attributes of window component is also added in $D_{update}$.

### Consensus algorithm used in the model

Raft^59^ is used as the consensus mechanism of BC in this paper. It is a distributed consensus mechanism, which is widely used in consortium blockchains. Raft is mainly used to manage the consistency of log replication which can solve the problem of information synchronization in the multi-person collaborative design of BIM drawing. Meanwhile, Raft can tolerate 50% non-Byzantine nodes, and using this mechanism can improve the stability of the multi-person collaborative design model of BIM drawing.

There are 3 roles in the nodes of Raft: the follower, the candidate, and the leader. The consensus process includes leader election and log replication. In this paper, the consensus process is described as follows:

1. Leader election: This election is random. BIM_O or BIM_D nodes can be elected as leader. Only leader can process requests initiated by BIM_O or BIM_D. Followers will forward the requests to leader for processing after receiving requests, while candidates will directly reject.
2. Log replication: ➀ BIM_O or BIM_D sends a request for obtaining or uploading BIM drawing data to the leader, and the leader processes request and sends to other BIM_O and BIM_D nodes, waiting to receive responses from BIM_O and BIM_D nodes; ➁ consensus is reached when leader received the confirmation responses from most nodes, leader first executes the request on the local ledger, and then informs other nodes that the consensus has reached; ➂ after receiving the notification of "consensus reached" from leader, other nodes also execute the request in the local ledger, and finally achieve the consistency of the request, that means reach the consensus.

## Experimental verification and evaluation analysis

Firstly, this section compares and analyzes the proposed scheme with existing blockchain-based scheme of BIM collaborative design. Subsequently, the performance and consistency of BIM drawing in the model are tested and evaluated through multi-machine experimental environment. The safety and reliability of data in the model is guaranteed by the characteristics of blockchain itself. The problem of redundancy of BIM drawing design information has been tested and analyzed in literature^25^ and will not be repeated here. At the end of this section, the security and reliability of proposed model are evaluated, and an actual scenario is used as an example to verify the effectiveness of the multi-person collaborative design model proposed in this paper.

### Scheme comparison

The comparison between proposed scheme and other existing schemes is shown in Table 1.

Tao et al.^22^ adopted the storage method combining on-chain and off-chain to reduce the storage pressure of blockchain. With only storing the summary of BIM design operation information in blockchain, accurate design content cannot be provided, and thus it cannot be used as an effective copyright proof. Therefore, proposed scheme and literatures^24,25^ stored the BIM design content in blockchain to protect designer’s copyright interests. However, literatures^24,25^ have not yet effectively integrated blockchain and BIM during the process of BIM collaborative design, and have not yet addressed issues such as the consistency of BIM drawing versions and the management of safety and reliability.

Although Shen et al.^23^ solved the problem of BIM version conflict, their solution is inefficient. Besides, their centralized off-chain storage method has security risks such as single-point failure and data tampering. The storage of large amount of useless design data also leads to information redundancy in blockchain. In response to the above problems in literature^23^, this paper uses blockchain and IPFS to realize the collaborative storage of data, so as to ensure the safety and reliability of data (see section “Security and reliability analysis”). In addition, SDT method and cycle division mechanism are used to realize the incremental update of BIM drawing and reduce information redundancy, which ensure the consistency of BIM drawing and improve the collaborative design efficiency.

### Multi-machine experimental environment configuration of the system

The prototype system is built by using the Hyperledger Fabric 1.4 framework.

The experimental equipment is a laptop and two Raspberry Pi 4B. The laptop has 16 GB of memory, an 8-Core Intel Core i7 processor, and three virtual machines with 2 Cores and 2 GB of memory. The virtual machines are all equipped with the Ubuntu 18.04 system. The two Raspberry Pi 4B both have 2 GB of memory, a 4-core Broadcom processor, and are equipped with an Ubuntu 18.04 arm version system. The Raft experimental environment has 5 Orderer Services, 5 organizations, and 5 Peer nodes. Each organization has a Certification Authority (CA) Service and a Peer node. Each node corresponds to a CouchDB. The endorsement policy is set to at least one endorsing node in each organization to participate in the endorsement.

### System experiment environment deployment

**Figure 13:**
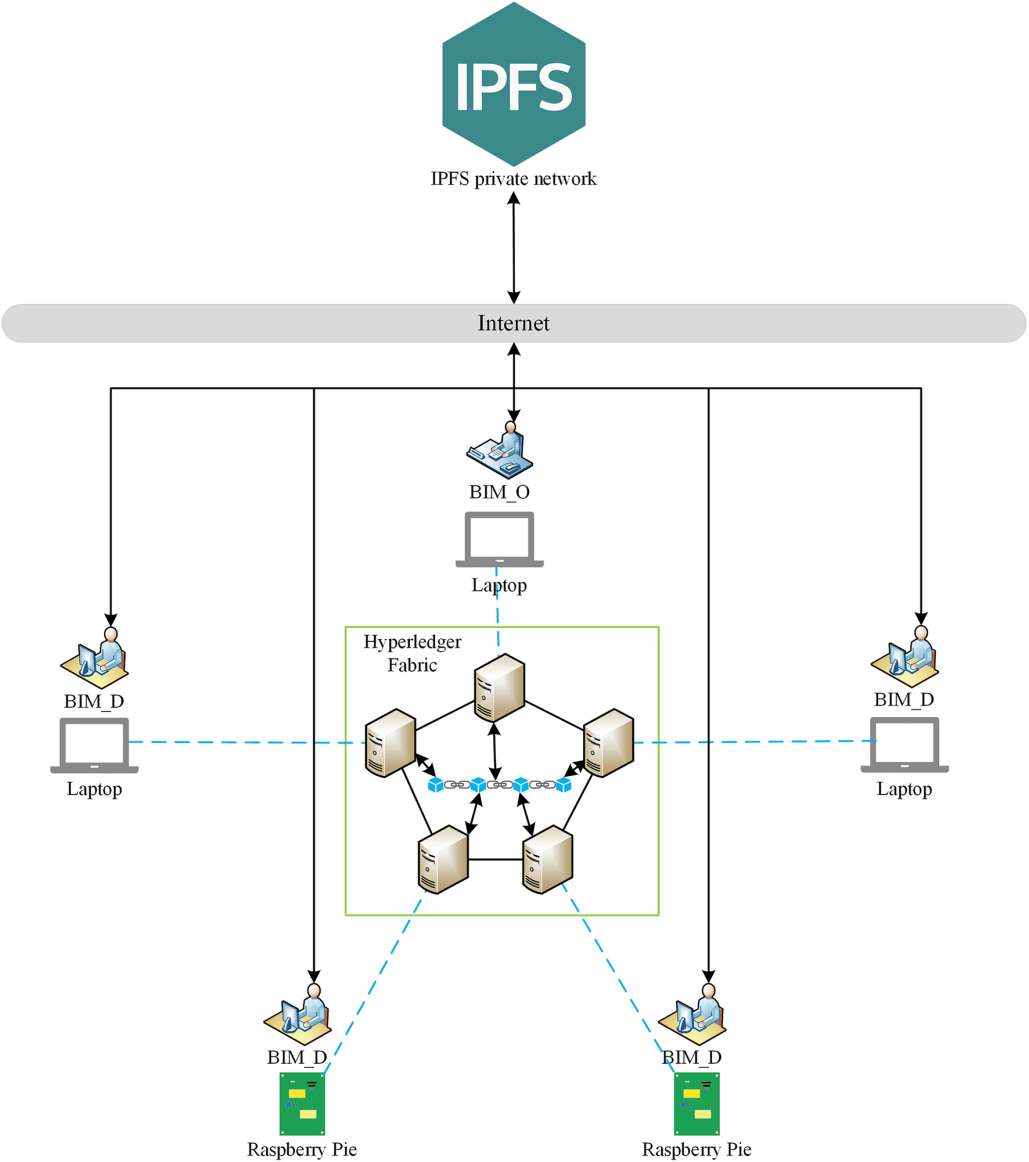
System experiment environment deployment.

The deployment of the system experiment environment is shown in Fig. 13. Use the virtual machine client in a laptop or Raspberry Pi to simulate a total of five users of BIM_O and BIM_D. Blockchain nodes are deployed in virtual machines, and all blockchain nodes are connected to each other to form a Hyperledger Fabric blockchain network. There is a total of one application channel and five organizations in the network, and each organization has one user. BIM_O and BIM_D exchange BIM drawing data with the Hyperledger Fabric blockchain network through the blockchain node in the corresponding virtual machine. At the same time, BIM_O and BIM_D are also building IPFS nodes locally and building an IPFS private network to upload and download BIM drawing.

### System performance evaluation

The BIM drawing used for performance test is a health care center in Jiangxi Province, China. This center covers an area of about 50,000 square meters, with 5 floors, and the size of the drawing is 56 MB. According to literature^23^, the test focuses on design time-consuming and throughput. Test results of proposed scheme are compared with Shen et al.^23^. Among them, design time-consuming includes storage/acquisition time of BIM drawing, execution time of contract. They are used to evaluate the design efficiency of proposed scheme. Throughput is used to evaluate the concurrent efficiency of proposed scheme.

#### Design time-consuming assessment

In the built multi-machine experimental environment, automated script is written to test storage/acquisition time of BIM drawing and execution time of contract in each cycle of proposed scheme (designing, updating the drawing or other related operations via local software are not influenced by proposed scheme itself, so the related operations will not be discussed here). Test results are compared with literature^23^, as shown in Table 4. Among them: (1) the operation of drawing storage and acquisition are implemented based on IPFS in proposed scheme, while it is implemented based on centralized cloud server^23^; (2) drawing update information in proposed scheme represents the $DDI$ calculated by BIM_D through SDT method, while it represents the complete information summary of BIM drawing after design^23^.

**Table 4:** Time-consuming comparisons of drawing storage, acquisition and contract execution.

		Proposed scheme	Literature^23^
Drawing review and upload cycle	Storage of drawing (off-chain)	27,847 ms	26,703 ms
	Uploading drawing summary information to blockchain (contract)	710 ms	747 ms
Drawing design cycle	Acquisition of drawing (off-chain)	1906 ms	26,159 ms
	Uploading drawing update information to blockchain (contract)	896 ms	728 ms
Drawing design information fusion cycle	Fusing drawing design information (contract)	7786 ms	
Total time (single-round design)		39,145 ms	54,337 ms

It can be seen from Table 4 that the acquisition efficiency of drawing is better than that of literature^23^, while the drawing storage efficiency is slightly lower. BIM_O in proposed scheme needs to synchronize BIM drawing to other BIM_D through IPFS network when storing the drawing, so it takes longer than only storing the drawing to cloud^23^. When acquiring the drawing, BIM_D in proposed scheme can directly obtain the corresponding drawing in local IPFS repository according to the storage address, therefore, the acquisition efficiency is high. However, in literature^23^, remote request needs to be sent to cloud server for acquiring drawing, which takes a long time in data transmission.

In terms of contract execution, since proposed scheme needs to upload $DDI$ to blockchain, execution time of storing drawing update information is higher than that in literature^23^ (only stores summary or other modification logs). In addition, proposed scheme designs an additional contract $DFDI\_C$ to fuse multiple $DDI$, and the fusion result is available for BIM_O to update BIM drawing locally. Based on the above operation, proposed scheme can realize the incremental update of BIM drawing, reduce information redundancy and reduce the number of times that BIM_O updates drawing locally, thereby improving the efficiency of collaborative design.

The test results show that time-consuming of single-round collaborative design in proposed scheme is better than that in literature^23^, which proves that proposed scheme can effectively reduce the design time-consuming of BIM drawing. Besides, based on the characteristics of IPFS, proposed scheme also ensures the reliability of BIM drawing and low redundancy (section “Security and reliability analysis” analyzes the reliability of BIM drawing).

#### Throughput assessment

Considering the scenario that multiple BIM_D initiate a large number of design transactions within a period of time, transaction throughput of prototype system is tested based on Hyperledger Caliper (performance test framework for Hyperledger Fabric). Maximum transaction throughput of blockchain system is tested when processing a large number of design transactions sent by multiple BIM_D per unit time. The test environment is set as follows: 4 BIM_D nodes concurrently initiate a total of 400, 600, 800, 1000, 1200, 1400, 1600, and 1800 $DDI$ uploading transactions per unit time. The maximum transaction throughput of blockchain system is tested, and the results are compared with literature^23^.

**Figure 14:**
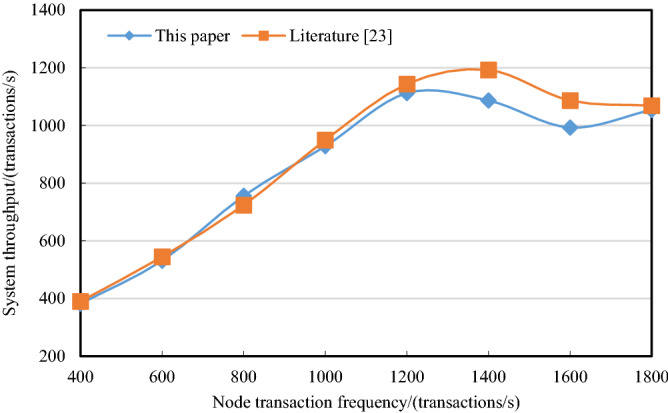
Comparison of system transaction throughput.

The results are shown in Fig. 14. Due to the same experimental environment and no changes of Fabric architecture, the maximum throughput of proposed scheme and literature^23^ are 1112 transactions per second and 1193 transactions per second respectively. The performance difference of throughput can be approximately ignored. In addition, requirements of performance in actual multi-person collaborative design are far less than test results. Therefore, performance of proposed scheme can meet the daily requirements of BIM_D and system, which can ensure the availability and effectiveness of proposed scheme.

#### Performance analysis of BIM drawing update operation

In view of the scenario where BIM_O updates multiple BIM drawing during the drawing review upload cycle, the performance of the BIM drawing update operation is analyzed. The performance is related to the time complexity of the BIM drawing update algorithm. The following focuses on the analysis of the time complexity of the BIM drawing update algorithm.

The BIM drawing update algorithm completes the update through two traversals. First, it traverses $DFDI$ to obtain the hierarchical relationship of the element attributes of the BIM drawing component to be updated, and then traverses the content of the BIM drawing before the update to find and update the attribute of the BIM drawing component corresponding to the hierarchical relationship. Among them, the update operation includes modification, addition, or deletion, and is executed only once, and the time complexity is O(*1*). Therefore, the time complexity of the BIM drawing update algorithm mainly depends on the time complexity of the traversal operation.

Recursively traverse $DFDI$ and BIM drawing content before updating. Set the BIM drawing component element attribute levels of the $DFDI$ and the BIM drawing content before the update to *m* and *n* and *m* > *n*, then the time complexity of the two traversals are O(*m*) and O(*n*) respectively. The time complexity of the BIM drawing update algorithm depends on the larger one of *m* and *n*, and the time complexity is linear time complexity O(*m*). In summary, the BIM drawing update operation performance is better, and it does not affect the overall operating efficiency of the system.

### Test of BIM drawing consistency

The model proposed in this paper ensures that BIM drawing obtained by BIM_D from IPFS can remain consistent after multiple rounds of design, that is, obtained BIM drawing have the same content. A round of design refers to starting from the drawing review upload cycle, after the drawing design cycle and the drawing design information integration cycle, and then returning to the drawing review upload cycle. The test is achieved by writing test scripts. The test environment is set as follows: 4 BIM_D perform 1,000 rounds of design on the initial blank BIM drawing. Each round must ensure that at least one BIM_D designs the drawing. The design content is not limited but must be quite different from the pre-design drawing. The purpose of test is to verify whether the contents of BIM drawing obtained by different BIM_D from IPFS are consistent.

**Figure 15:**
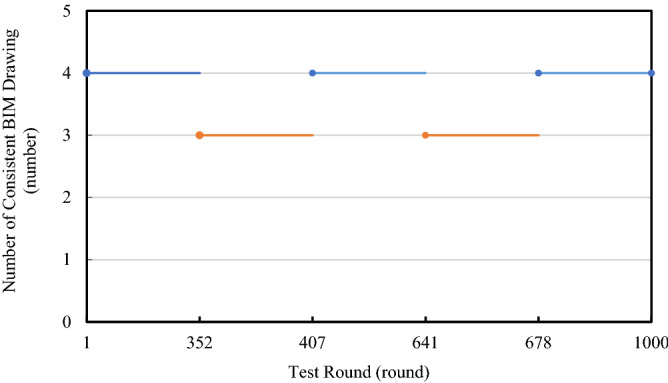
BIM drawing consistency.

The test results are shown in Fig. 15. During rounds 352 to 406 and 641 to 677, the number of consistent BIM drawing decreases to 3. The inconsistency of drawing is caused by faults such as disconnection and downtime of individual nodes. After the node recovers from fault, the number of consistent BIM drawing becomes 4 again. The experimental results show that proposed model has good robustness. In addition, proposed model can ensure that all BIM_D design on the same BIM drawing when no faulty node exists, thereby meeting the design requirements for ensuring the consistency of BIM drawings during the process of multi-person collaborative design.

### Security and reliability analysis

This section analyzes the multi-person collaborative design model proposed in this paper from the perspective of security and reliability. The analysis contents include:

1. User permission problem. BIM_O can set the modification right of the BIM drawing when initializing the BIM drawing, and specify which user (BIM_O) has the right to participate in the design of the BIM drawing (edit/modify/delete related data components in the drawing). In addition, BIM_O can dynamically update the modification right list in the subsequent creation process to avoid malicious tampering of the BIM drawing content by illegal users, thereby ensuring the normal operation of the multi-person collaborative design process.
2. The problem of key loss. In the process of multi-person collaborative design of BIM drawing, the problem of key loss may occur: ➀ If BIM_D loses the private key, BIM_D can re-apply for the public and private key from the CA. After proving BIM_D’s identity offline, BIM_O updates the list of modification rights, adds the new public key of BIM_D to it, and removes the original public key; ➁ If BIM_O loses the private key, after reapplying for the public and private keys, BIM_O needs to negotiate with all BIM_D offline to prove identity, and distribute the new public key to each BIM_D. In the subsequent collaborative design process, BIM_D uses the new public key to verify the signature and identity of BIM_O.
3. The reliability of BIM drawing. In this model, the reliability of BIM drawing stored in IPFS and drawing design information stored in BC is guaranteed by the cryptographic algorithm and distributed storage features within IPFS and BC. BIM_D or BIM_O firstly judges the validity and authenticity of BIM drawing and related information by verifying signatures before designing and updating drawing. Then operate on the basis of valid BIM drawing or related information that have passed the verification, so as to ensure the reliability of BIM drawing in the entire collaborative design process.

### Illustration example

In order to further illustrate the effectiveness of the model proposed in this paper, this section takes the modification of the wall in the BIM drawing of the health care center as an example to introduce the multi-person collaborative design process of BIM_O and BIM_D in the constructed prototype system.

#### Step1: BIM_O creates initial BIM drawing

**Figure 16:**
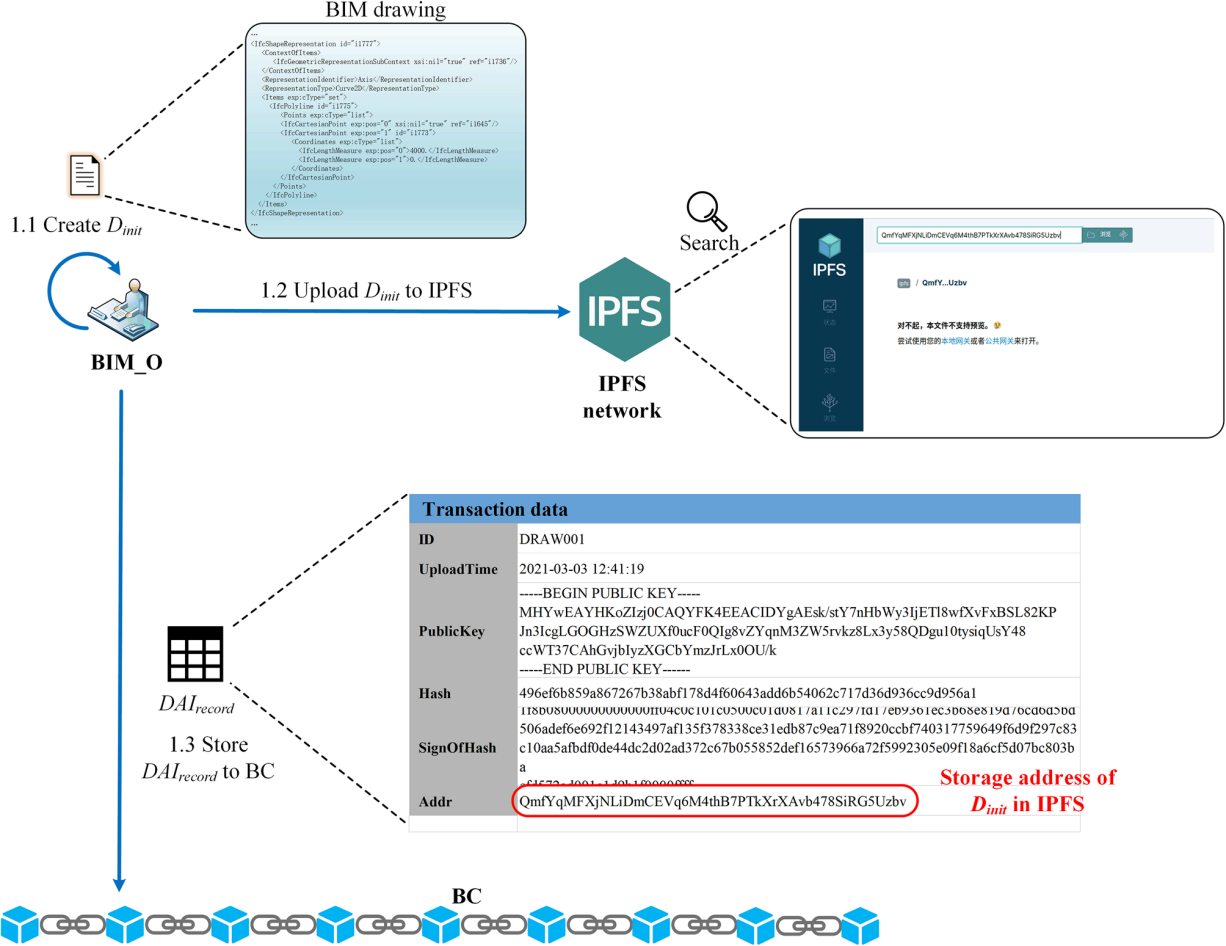
Example: BIM_O initializes BIM drawing.

**Figure 17:**
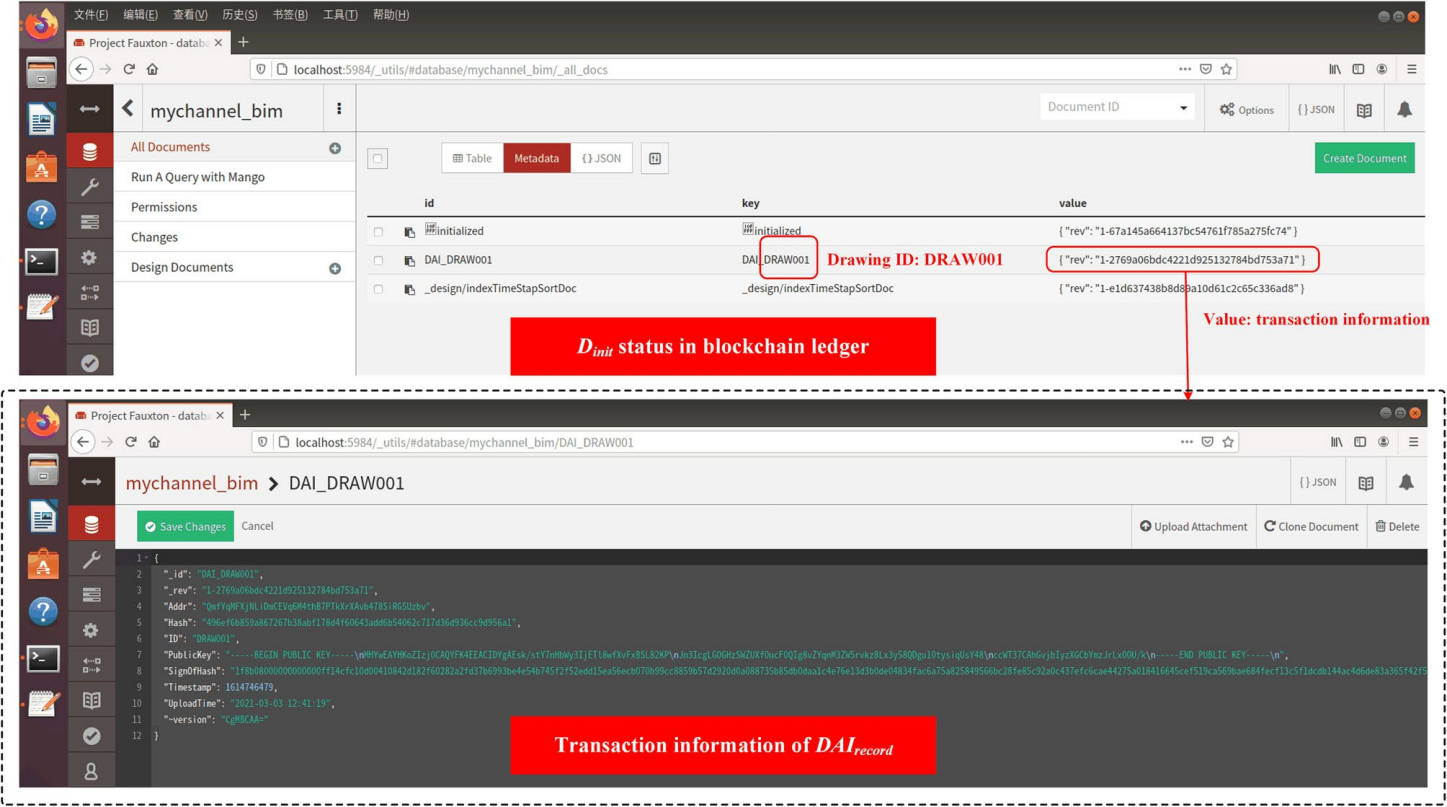
BIM drawing summary information in blockchain ledger.

As shown in Fig. 16, in the drawing review and upload cycle, BIM_O first creates $D_{init}$, then uploads it to the IPFS network, and then obtains the returned $Addr_{IPFS}$ (e.g., "QmfYqMFX…iRG5Uzbv" in Fig. 16). After that, construct $DAI_{record}$ and store it into BC. Figure 17 shows the specific transaction information of $DAI_{record}$ in the BC ledger (CouchDB), where "DRAW001" represents the ID number of the drawing.

#### Step2: BIM_D design BIM drawing

**Figure 18:**
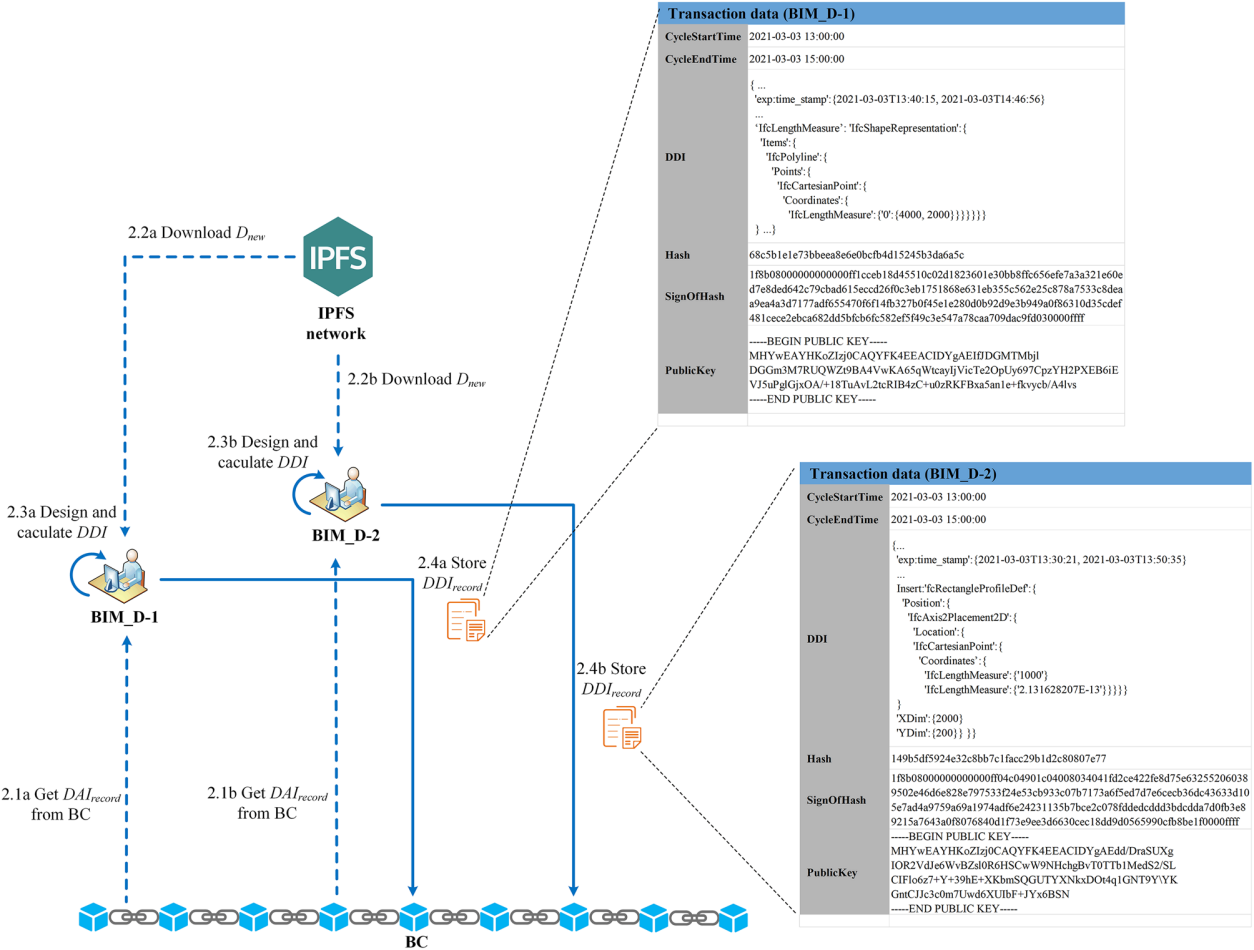
Example: BIM_D design BIM drawing.

**Figure 19:**
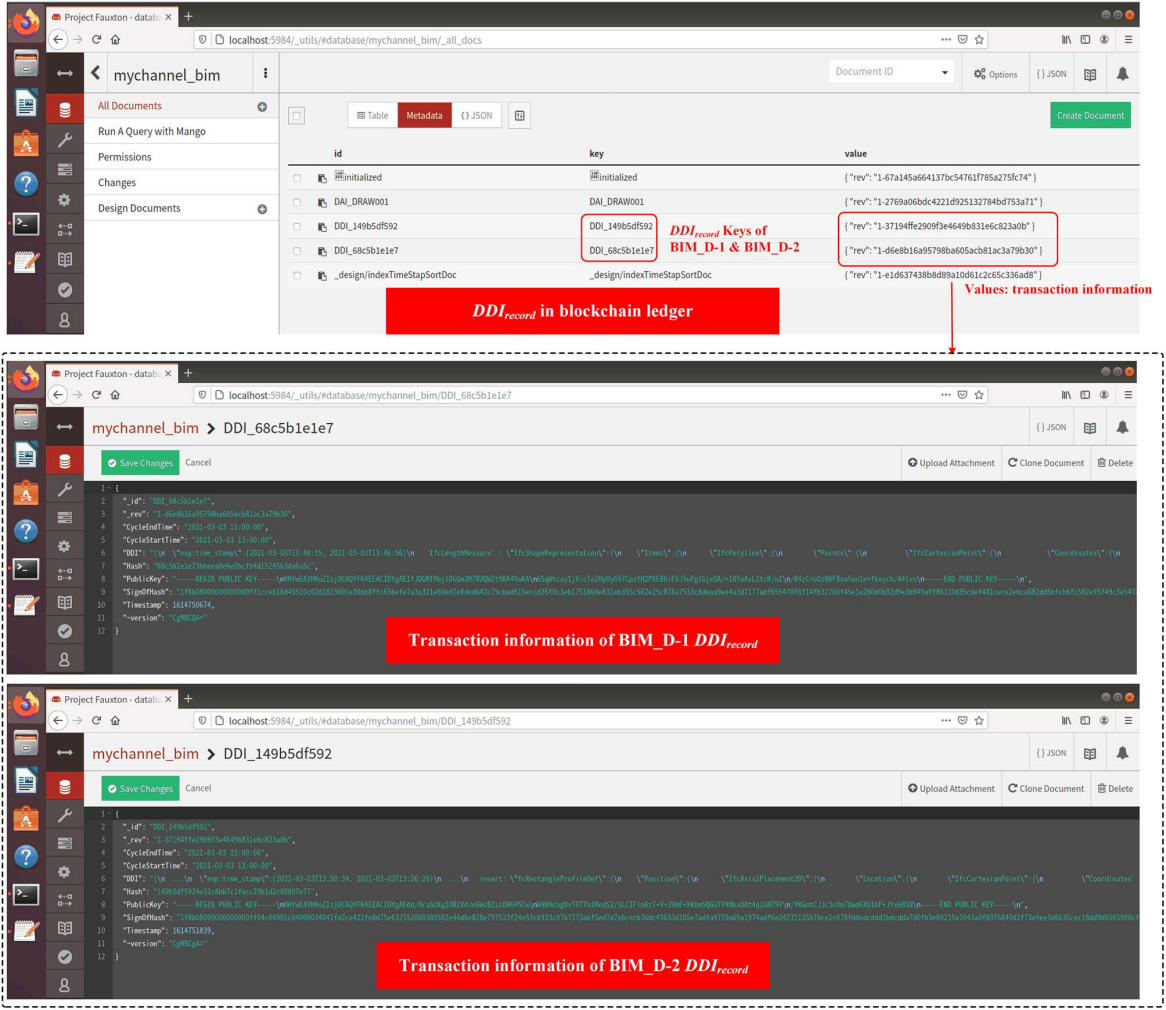
BIM drawing design information in blockchain ledger.

BIM_D-1 intends to modify the width of the wall in the BIM drawing, and BIM_D-2 intends to add a window on the wall. The design flow is shown in Fig. 18. At different time points in the same drawing design cycle (the design time of each round is fixed at 2 h, the start time of this round is 2021–03-03 13:00:00, and the end time is 2021-03-03 15:00: 00), they get the summary information of the drawing numbered "DRAW001" from BC, and download the latest BIM drawing from IPFS according to $Addr_{IPFS}$. Subsequently, they each carry out the drawing design locally, and calculate the corresponding $DDI$ based on the SDT method after the design was completed. Finally, BIM_D-1 and BIM_D-2 call the smart contract to construct $DDI_{record}$ at different time points and store it in the BC ledger (as shown in Fig. 19).

#### Step3: BIM_O update BIM drawing

**Figure 20:**
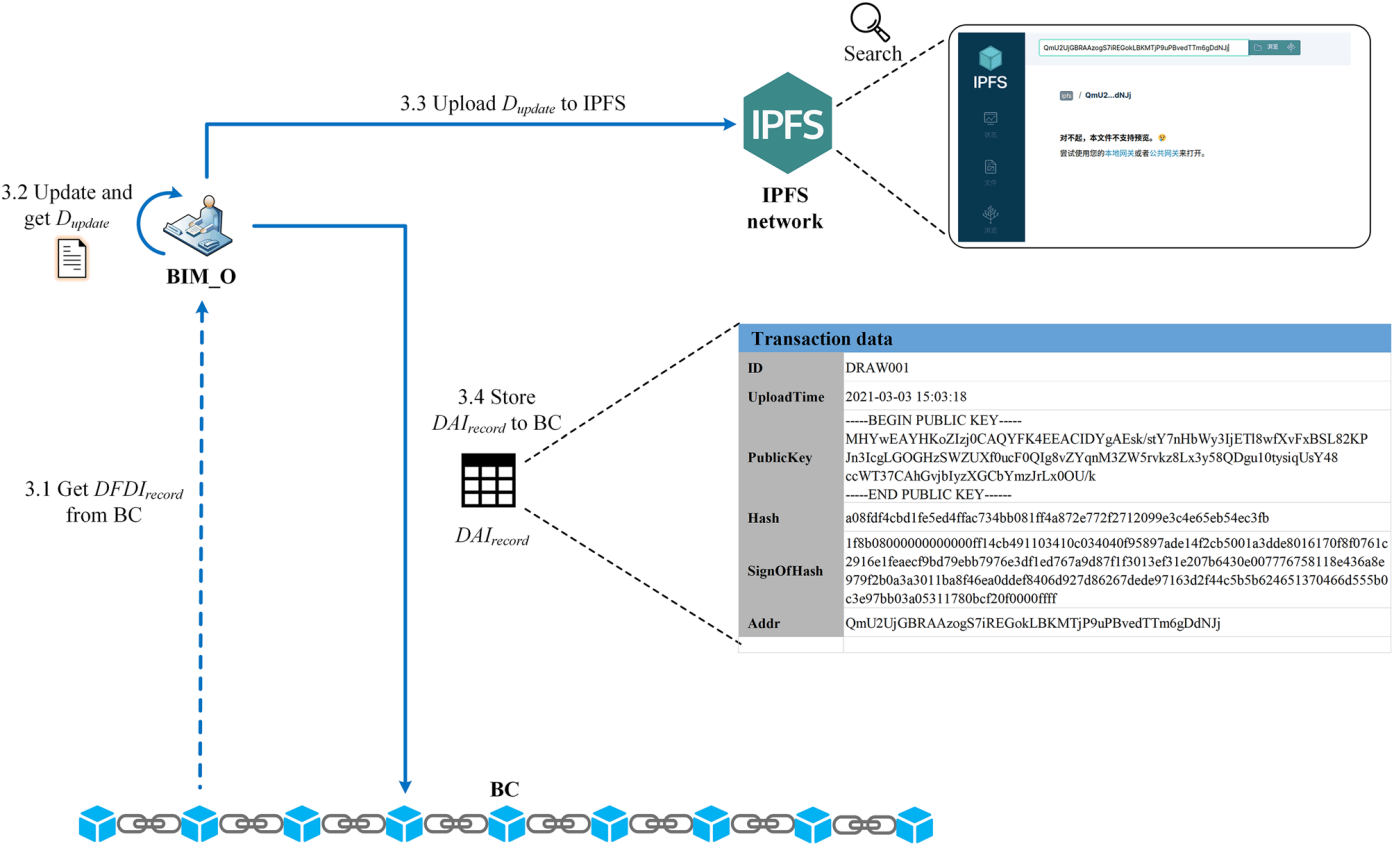
Example: BIM_O update BIM drawing.

Figure 20 shows the process of BIM_O updating BIM drawing. After the drawing design cycle ends, it will enter the drawing design information fusion cycle. In the drawing design information fusion cycle, BC will automatically execute $DDI\_C_{download}$ to fuse all $DDI$ to generate $DFDI$. BIM_O calls the smart contract to obtain $DFDI$ from BC, and then uses $DFDI$ to update the local drawing content to get $D_{update}$. Finally, BIM_O uploads $D_{update}$ to the IPFS network, then creates $DAI_{record}$ and stores it in BC.

At this point, the multi-person collaborative design process of BIM drawing is over. If BIM_D or BIM_O still has other design requirements, BIM_D and BIM_O will repeat the steps in sections “Step2: BIM_D design BIM drawing” and “Step3: BIM_O update BIM drawing” until BIM_O terminates the design of BIM drawing.

## Conclusion

In large-scale construction projects, multi-person collaborative design of BIM drawing is very important, which can greatly improve the efficiency of BIM drawing design, accelerate the construction of construction projects, and promote the continuous development of construction field. In order to solve the problems of poor data security and reliability, inconsistent BIM drawing information, information redundancy, and inaccurate protection of copyright interests in the existing multi-person collaborative design methods of BIM drawing, this paper proposes a multi-person collaborative design model for BIM drawing based on the collaborative storage of blockchain (on-chain) and IPFS (off-chain). This model stores the encrypted drawing data through a collaborative method, which not only ensures the security, reliability and integrity of data, but also improves the scalability of blockchain. Besides, this model uses a period division mechanism to avoid the problem of information synchronization during the process of BIM drawing design. It uses the SDT method to achieve incremental updates of BIM drawing, reduce information redundancy, and uses blockchain to record the designer's incremental design information to provide designers with the accurate copyright basis. Finally, the experiment is designed to test the performance and security of proposed scheme, and the example is used to demonstrate the usability of proposed scheme.

Although this paper has built an efficient and usable multi-person collaborative design model of BIM drawing based on blockchain, it also has some shortcomings. The step of BIM drawing design information fusion in the model takes extra time to complete, which will have a certain impact on the model efficiency. Therefore, in future research work, we consider combining the fusion process of BIM drawing design information with the consensus process of blockchain to complete the information fusion in consensus process and avoid additional time overhead. In addition, the performance of SDT method needs to be advanced to further improve the efficiency of handling large-scale BIM drawing information.

## Data Availability

The datasets generated during and/or analyzed during the current study are available from the corresponding author on reasonable request.
